# Prediction of clinically significant prostate cancer through urine metabolomic signatures: A large-scale validated study

**DOI:** 10.1186/s12967-023-04424-9

**Published:** 2023-10-11

**Authors:** Hsiang-Po Huang, Chung-Hsin Chen, Kai-Hsiung Chang, Ming-Shyue Lee, Cheng-Fan Lee, Yen-Hsiang Chao, Shih-Yu Lu, Tzu-Fan Wu, Sung-Tzu Liang, Chih-Yu Lin, Yuan Chi Lin, Shih-Ping Liu, Yu-Chuan Lu, Chia-Tung Shun, William J. Huang, Tzu-Ping Lin, Ming-Hsuan Ku, Hsiao-Jen Chung, Yen-Hwa Chang, Chun-Hou Liao, Chih-Chin Yu, Shiu-Dong Chung, Yao-Chou Tsai, Chia-Chang Wu, Kuan-Chou Chen, Chen-Hsun Ho, Pei-Wen Hsiao, Yeong-Shiau Pu

**Affiliations:** 1 Graduate Institute of Medical Genomics and Proteomics, College of Medicine, National Taiwan University, Taipei, Taiwan; 2 Department of Urology, National Taiwan University College of Medicine and Hospital, 7 Zhongshan South Road, Taipei, 100225 Taiwan, Republic of China; 3 Institute of Cellular and System Medicine, National Health Research Institutes, Miaoli, Taiwan; 4 Department of Biochemistry and Molecular Biology, College of Medicine, National Taiwan University, Taipei, Taiwan; 5 Agricultural Biotechnology Research Center, Academia Sinica, No. 128, Sec. 2, Academia Road, Nankang, Taipei, 11529 Taiwan; 6 Department of Medicine, College of Medicine, National Taiwan University, Taipei, Taiwan; 7 Department of Surgical Oncology, National Taiwan University Cancer Center, Taipei, Taiwan; 8 Department of Pathology, College of Medicine, National Taiwan University, Taipei, Taiwan; 9 Department of Urology, Taipei Veterans General Hospital, National Yang Ming Chiao Tung University, Taipei, Taiwan; 10 Division of Urology, Department of Surgery, Cardinal Tien Hospital, New Taipei City, Taiwan; 11 School of Medicine, College of Medicine, Fu-Jen Catholic University, New Taipei City, Taiwan; 12 Division of Urology, Department of Surgery, Taipei Tzu Chi Hospital, and the Buddhist Tzu Chi Medical Foundation, College of Medicine, Tzu Chi University, Hualien, Taiwan; 13 Division of Urology, Department of Surgery, Far Eastern Memorial Hospital, and Department of Nursing, College of Healthcare & Management, Asia Eastern University of Science and Technology, New Taipei City, Taiwan; 14 Department of Medicine & Division of Urology, Taipei Tzu Chi Hospital, New Taipei City, Taiwan; 15 Department of Urology, School of Medicine, College of Medicine, Taipei Medical University, Taipei, Taiwan; 16 Department of Urology, Shuang-Ho Hospital, Taipei Medical University, New Taipei City, Taiwan; 17 TMU Research Center of Urology and Kidney, Taipei Medical University, Taipei, Taiwan; 18 Graduate Institute of Clinical Medicine, Taipei Medical University, Taipei, Taiwan; 19 Division of Urology, Department of Surgery, Shin Kong Wu Ho-Su Memorial Hospital, Taipei, Taiwan

**Keywords:** Prediction, Aggressive, Liquid biopsy, Active surveillance, Diagnosis

## Abstract

**Purpose:**

Currently, there are no accurate markers for predicting potentially lethal prostate cancer (PC) before biopsy. This study aimed to develop urine tests to predict clinically significant PC (sPC) in men at risk.

**Methods:**

Urine samples from 928 men, namely, 660 PC patients and 268 benign subjects, were analyzed by gas chromatography/quadrupole time-of-flight mass spectrophotometry (GC/Q-TOF MS) metabolomic profiling to construct four predictive models. Model I discriminated between PC and benign cases. Models II, III, and GS, respectively, predicted sPC in those classified as having favorable intermediate risk or higher, unfavorable intermediate risk or higher (according to the National Comprehensive Cancer Network risk groupings), and a Gleason sum (GS) of ≥ 7. Multivariable logistic regression was used to evaluate the area under the receiver operating characteristic curves (AUC).

**Results:**

In Models I, II, III, and GS, the best AUCs (0.94, 0.85, 0.82, and 0.80, respectively; training cohort, N = 603) involved 26, 24, 26, and 22 metabolites, respectively. The addition of five clinical risk factors (serum prostate-specific antigen, patient age, previous negative biopsy, digital rectal examination, and family history) significantly improved the AUCs of the models (0.95, 0.92, 0.92, and 0.87, respectively). At 90% sensitivity, 48%, 47%, 50%, and 36% of unnecessary biopsies could be avoided. These models were successfully validated against an independent validation cohort (N = 325). Decision curve analysis showed a significant clinical net benefit with each combined model at low threshold probabilities. Models II and III were more robust and clinically relevant than Model GS.

**Conclusion:**

This urine test, which combines urine metabolic markers and clinical factors, may be used to predict sPC and thereby inform the necessity of biopsy in men with an elevated PC risk.

**Supplementary Information:**

The online version contains supplementary material available at 10.1186/s12967-023-04424-9.

## Introduction

Prostate cancer (PC) is a significant global health issue. In 2020, it affected over 1.4 million men and caused mortality in over 0.3 million men [1]. In the United States, the age-standardized incidence of PC was 106.4 per 100,000 population [2]. The diagnosis of PC depends on histopathological examination of prostate tissue samples obtained during biopsy or surgery. Treatment for PC is typically guided by key clinicopathological factors [3], including serum prostate-specific antigen (PSA) levels, clinical staging, biopsy Gleason sum (GS), patient age, and co-morbidities, as documented in the National Comprehensive Cancer Network (NCCN) guidelines [4] and the European Association of Urology guidelines [5]. Generally, localized or nonmetastatic disease is either treated definitively for potentially lethal or clinically significant PC (sPC) or conservatively for indolent or insignificant PC (isPC), depending on tumor aggressiveness [3, 4, 6]. The prognosis of PC is more favorable than that of other types of cancer [2]. Nevertheless, the 5-year survival rates for PC depend on the stage: > 99% for the localized nonmetastatic stage, 31% for the distant stage, and 98% for all stages combined [2]. Multiple factors can influence the prognosis of patients, including the tumor stage and grade at primary diagnosis, and the patient's age and overall health [6]. Unfortunately, the GS of the tumor is unknown before prostate biopsy and staging involves postbiopsy advanced imaging modalities. Therefore, avoiding unnecessary biopsy and overdiagnosis using noninvasive tests that accurately predict tumor aggressiveness in men with an elevated risk of PC is an unmet clinical need [7].

Serum PSA is a powerful screening marker [8] and has helped reduce the metastatic PCs and mortality in PC patients [9, 10]; however, the PSA test for PC prediction lacks a balance between sensitivity and specificity to the extent that when one aspect reaches 80%, the other decreases to 30% [11]. Thus, high false-positive rates are an issue faced by clinicians [12, 13]. Moreover, the test poorly differentiates sPC from isPC at levels below 10–20 ng/mL [14, 15]. Most experts recognize that PSA testing increases the risk of overdetection of otherwise indolent diseases and the consequential risk of overtreatment, which may potentially expose patients to anxiety and treatment-related morbidities [13]. Therefore, the NCCN guidelines emphasize using more techniques and biomarker tests, if available, to optimize the detection of sPC while minimizing the identification of those with isPC [13]. Generally, sPC is regarded as an aggressive and potentially lethal PC, usually characterized by higher PSA levels, higher GSs, and more advanced clinical stages [3, 13]. In contrast, isPC is rarely lethal and exhibits lower PSA levels, GSs, and/or clinical stages. In men with elevated PSA levels, although the biopsy GS may reveal cancer aggressiveness [16], a postbiopsy staging workup remains necessary to inform treatment planning [4, 17]. Furthermore, a convenient, accurate, and robust test to differentiate sPC from isPC before a biopsy is currently unavailable.

Many clinical risk factor-based prediction models [18, 19] are used to predict PC or high-grade PC (GS ≥ 7) in men at risk. These models incorporate serum PSA level, patient age, race, family history, previous biopsy results, and digital rectal examination (DRE) findings, which usually have area under the receiver operating characteristic (ROC) curve (AUC) values ranging from 0.61 to 0.77 [18, 20, 21]. Notably, almost all these models define GS ≥ 7 as indicative of sPC and neglect tumor staging information, which may be less optimal than complete risk stratification. Examples of such models include the D’Amico classification [22] and the NCCN risk groupings [4], which are based on PSA levels, GSs, and clinical stages.

Multiparametric magnetic resonance imaging (mpMRI) has emerged as an important diagnostic tool for PC [23]. In recent guidelines, it is recommended for routine use in men with elevated PSA levels to inform the need for biopsy and reduce unnecessary biopsies [24, 25]; however, the AUCs of mpMRI for predicting sPC ranged only from 0.79 to 0.84 [26]. Ideally, a test that predicts sPC should be more accurate and noninvasive, utilize samples that are easy to obtain (such as urine), and be predictive of complete risk stratification instead of the GS only.

In the era of precision medicine, liquid biopsies have uncovered useful biomarkers that facilitate the diagnosis and stratification of various cancers [27]. Liquid biopsy markers, such as the Prostate Health Index [28], 4 K score [29], urine RNA PCA3 [30], and SelectMDx [31] have been successfully used to estimate the risk of PC or sPC [32–34]. Moreover, liquid biopsies have emerged as a valuable tool for improving PC management by predicting castration-resistant PC prognosis [35], evaluating patients’ drug response [36], and identifying candidates for targeted therapy [37]. Urine metabolomics has been used in biomarker detection for not only urinary tract cancers but also other cancers [27]. Owing to its noninvasive nature and the close anatomical proximity between the prostate and the urinary tract, urine metabolomics presents unique advantages over other liquid biopsies in PC. Over the past decade urine metabolomics has been investigated in biomarker studies for PC or sPC [38, 39]. Moreover, the urine metabolome recapitulates some dysregulated metabolic pathways in PC [39], suggesting its potential application in other clinical contexts, such as predicting treatment response. However, most urine metabolomic studies have aimed to differentiate benign subjects from those with PC [39]. Few studies have focused on discriminating isPC from sPC, and those that have were primarily proof-of-concept or small-sample (e.g., less than 80) studies and lacked validation [40, 41]. In this large-scale study, we demonstrated that the combination of urine metabolic marker panels and clinical risk factors can differentiate benign cases from cancer and isPC from sPC with very high accuracy. Thus, our models may greatly assist clinical decision-making before biopsy for men at risk.

## Materials and methods

### Subject enrollment and eligibility criteria

Between August 2017 and April 2021, 893 men with an elevated risk of PC at eight hospitals (the BigUro Study Team) in Taiwan were enrolled before the prostate biopsy. Another group of 258 men with newly diagnosed treatment-naïve PC was enrolled at least six weeks after the biopsy to mitigate post-biopsy changes in urine omics profiling. All patients were ethnic Chinese men in Taiwan. The inclusion criteria were as follows: men ≥ 20 years old; PSA ≥ 4.0 ng/ml (with or without abnormal DRE); willingness to undergo prostate biopsy (for men without the diagnosis of PC yet) or subjects with untreated PC; and willingness to sign the informed consent form. Men (N = 56) with atypical small acinar proliferation or high-grade prostatic intraepithelial neoplasia were excluded from the analysis. The study was approved by the institutional review board of each hospital and registered at Clinicaltrials.gov (NCT03237702). All men signed an informed consent form before enrollment.

Fifty milliliters of spot urine and clinical information were collected from the two groups of subjects (N = 1151). Urine samples were centrifuged at 2500 × g for 15 min at 4 °C to collect the supernatants, which were stored at − 80 °C before use. We added 100 U of urease to 100 μL urine samples (aliquoted from 50 mL urine) to deplete excess urea by incubating at 37 °C with mild shaking at 650 rpm for 1 h. Termination of urease activity and metabolite extraction were carried out by admixing 1 mL methanol with a vortex for 30 s, and precipitated proteins were removed via centrifugation at 13,200 rpm for 15 min at 4 °C. The supernatants were transferred to 2-mL microcentrifugation tubes and dried in SpeedVac concentrators. The dried metabolic extract was derivatized using bis (trimethylsilyl)-trifluoroacetamide containing 1% trimethylchlorosilane and analyzed via gas chromatography (GC)/mass spectrometry.

### Gas chromatography quadrupole time-of-flight mass spectrophotometry

The derivatized samples were analyzed using an Agilent 7890B GC system coupled with a 7250 quadrupole time-of-flight mass spectrometer (Q-TOF MS) equipped with electron ionization. Separation was performed on a Zorbax DB5-MS + 10 m Duragard capillary column (30 m × 0.25 mm × 0.25 mm, Agilent, California, USA). The GC temperature profile was held at 60 °C for 1 min, raised to 325 °C at 10 °C/min, and held at 325 °C for 10 min. The transfer line and the ion source temperature were set at 300 °C and 280 °C, respectively. The mass range monitored was from 50 to 600 Daltons. Mass spectra were compared against the NIST 2017, Fiehn, and Wiley Registry 11th Edition mass spectra libraries. Metabolites that appeared in more than 60% of samples from the same risk group of patients were included for further marker panel selection. Perfluorotributylamine was used as a calibration standard for GC. The personnel for specimen handling or data acquisition were blinded to the disease grouping.

### Biopsy and staging workup

Prostate biopsy was performed using ≥ 12-core transrectal and/or transperineal biopsy. All PC patients underwent a postbiopsy staging workup, including mpMRI and bone scans. The NCCN risk groups [4] were assigned to all PC patients, ranging from very low risk (VLR), low risk (LR), favorable intermediate risk (FIR), unfavorable intermediate risk (UIR), high risk (HR), very high risk (VHR), and metastatic PC (mPC). Clinical staging was based on a combined review of DRE and MRI, whichever was higher.

### Training and validation cohorts

The subjects were randomly divided into training and validation cohorts (Table 1). The former was used to build predictive models, while the latter was independent of the model construction. The percentage of benign subjects in the training cohort was lower than that in the entire subject pool, so each respective risk group was adequately represented during model construction. However, we restored the percentage of each risk group in the validation cohort to approximately that of the entire subject pool.

**Table 1:** Demographics and clinical characteristics of the training and validation cohorts

	Combined cohort (%) (n = 928)	Training cohort (%) (n = 603)	Validation cohort (%) (n = 325)
NCCN risk group subject no. (%)			
Benign			
VLR and LR	268 (28.9%)	110 (18.2%)	158 (48.6%)
FIR	100 (10.8%)	74 (12.3%)	26 (8.0%)
UIR	99 (10.7%)	74 (12.3%)	25 (7.7%)
HR and VHR	139 (15.0%)	105 (17.4%)	34 (10.5%)
mPC	268 (28.9%)	202 (33.5%)	66 (20.3%)
	54 (5.8%)	38 (6.3%)	16 (4.9%)
Age (years)			
Median (mean)	69.0 (69.3)	69.0 (69.4)	69.0 (69.0)
P for MWU test		0.534	
PSA (ng/mL)			
Median (IQR)	9.26 (5.89–18.49)	9.92 (6.13–20.16)	8.65 (5.69–13.93)
P for MWU test		0.005	
Serum creatinine (mg/dL)			
Median (IQR)	1.0 (0.9–1.1)	1.0 (0.9–1.1)	1.0 (0.87–1.1)
P for MWU test		0.850	
Family history of PC			
Yes (%)	72 (7.9)	53 (9)	19 (6)
No (%)	838 (92.1)	539 (91)	299 (94)
P for Chi-square		0.1126	
Previous negative biopsy			
Yes (%)	181 (22.1)	115 (21.3)	66 (23.7)
No (%)	637 (77.9)	424 (78.7)	213 (76.3)
P for Chi-square		0.4486	
Abnormal DRE			
Yes (%)	322 (35.3)	236 (39.9)	86(27)
No (%)	589 (64.7)	356 (60.1)	233 (73)
P for Chi-square		0.0001	

*VLR* very low risk, *LR* low risk, *FIR* favorable intermediate risk, *UIR* unfavorable intermediate risk, *HR* high risk, *VHR* very high risk, *mPC* metastatic prostate cancer, *MWU* Mann–Whitney U test, *IQR* interquartile range

### Predictive models

Three models (Models I–III) for predicting dichotomous endpoints, namely, benign versus cancerous cases (Model I) and isPC versus sPC (Models II and III), were designed. Model II applied to men with a long life expectancy (> 10–15 years), where VLR/LR disease was regarded as isPC and all other higher-risk groups (from FIR to mPC) were regarded as sPC. Model III applied to men with a shorter life expectancy (< 10–15 years) where VLR/LR and FIR were regarded as isPC [4] and all other higher-risk groups were regarded as sPC. For comparison with previously published studies, we constructed a fourth model (Model GS) to predict high-grade PC (GS ≥ 7) using the same subject pool.

### Statistical analysis and marker selection

The significance of differences in clinical characteristics between the training and validation cohorts was determined using the Chi-square test (age, PSA level, and creatinine level) and Mann–Whitney U test (positive family history, previous biopsy, and DRE result). The peak values of all metabolites identified in GC/Q-TOF MS were normalized by both urine creatinine (determined via LC‒MS/MS) and total peak area values to reduce interbatch variances [42]. To select markers, K-fold (K = 5) cross-validation was performed, with four subcohorts as the training set and one as the testing set. The process was repeated for five rounds, which generated five training and testing sets per round. In each training set, univariate logistic regression was applied to exclude metabolites with a *p* value of > 0.1. Backward elimination based on the Akaike information criterion (AIC) [43] was conducted, followed by a multivariable logistic regression to exclude metabolites with a *p* value of > 0.1. The remaining metabolites were applied to the five testing sets in the model construction and ROC generation. We produced 100 models from 20 rounds. All the above stepwise logistic regression procedures were conducted using R (version 4.1.2). The top 30 highest-recurring metabolites from the top 75% of models were selected for conducting another multivariable logistic regression. The marker number per model was based on cumulative AIC nadir scores (Additional file 1: Fig. S1), which were obtained using R (version 4.1.2), to balance model fit and complexity and avoid overfitting. Multivariable logistic regression and performance analyses (AUC, sensitivity and specificity at Youden's index threshold, accuracy, and so on) were performed using both MedCalc (MedCalc Software Ltd., Ostend, Belgium) and R (version 4.1.2). Both the calculation of the *p* value for the AUC and the comparison of the AUCs of the two ROC curves were conducted according to the previously described method [44] using MedCalc. Decision curve analysis (DCA) was performed as previously described using R (version 4.1.2) [45]. Heatmaps with hierarchical clustering were generated using Python’s Seaborn clustermap (https://github.com/mwaskom/seaborn/). Bubble plots, which were based on our logistic regression analyses, were created using the ggplot2 package in R (version 4.1.2).

## Results

**Fig. 1:**
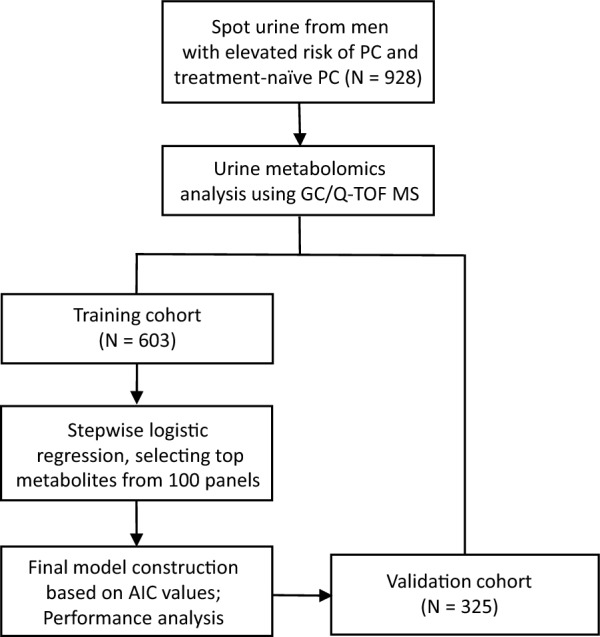
A flowchart of this study. Four predictive models derived from the training cohort were validated using an independent validation cohort. AIC: Akaike information criterion

The study flowchart is presented in Fig. 1.

### Baseline characteristics

Among all urine samples from the 1151 subjects, 928 samples, namely, 660 samples from PC patients and 268 randomly selected samples from 435 benign subjects, were sent for GC/Q-TOF MS. The median age of the subjects was 69 years. The characteristics of the training and validation cohorts are shown in Table 1. The validation cohort contained a percentage (48.8%) of benign cases similar to that in the original 1,151-subject pool, in which benign cases, VLR/LR, FIR, UIR, HR/VHR, and mPC accounted for 52.0%, 8.7%, 7.4%, 11%, 18.3%, and 2.6% of cases, respectively.

### Model performance

There were 1,941 identifiable metabolites in the GC/Q-TOF MS setting, of which, 172 outstanding metabolites fulfilled the filtering criteria for quality control and were included in the model construction. According to an AIC nadir search, 26, 24, 26, and 22 markers (Additional file 1: Fig. S1 and Table S1) were selected in the final panels for Models I, II, III, and GS, respectively, with AUCs of 0.94, 0.85, 0.82, and 0.80, respectively (training cohort, Table 2). When the five clinical risk factors—age, serum PSA, family history of PC, previous negative biopsy, and abnormal DRE—were added to the models, the combined Models I, II, III, and GS showed significantly improved AUCs (0.95, 0.92, and 0.92, and 0.87, respectively; all *p* < 0.0001), which were higher than those achieved by adding PSA alone to the models (0.94, 0.90, 0.90, and 0.85, respectively; all *p* < 0.05). The NCCN risk grouping-based Models II and III outperformed the GS-based Model GS. Model performance at 90% sensitivity is shown in Table 2 (training) and Table 3 (validation). Additional details regarding logistic regression parameters for panels and model performance at 95% sensitivity are provided in the supplementary information (Additional file 1: Table S2 and Table S3). Detailed properties of these markers are also listed in the supplementary information (Additional file 1: Table S1). Regarding the relative significance of the panel metabolites, bubble plots (Additional file 1: Fig. S2) revealed two major dysregulated metabolites: monopalmitin levels were frequently lower in the urine of cancer (Model I) or sPC (Models II, III, and GS) patients, while 1-stearoyl-rac-glycerol levels were higher in the urine of sPC (Models II, III, and GS) patients.

**Table 2:** Performance of the four predictive models (training cohort, 90% sensitivity)

	AUC (95% CI)	P	Sen (%)^a^	Spe (%)	NPV (%)	PPV (%)	Accuracy (%)	Bx avoided (%)^b^
	Model I: Benign vs Cancer (Marker number in model = 26)							
5 clinical factors^c^	0.75 (0.71–0.80)	< 10^–4^	90	30	40	85	79	16
Marker panel	0.94 (0.91–0.96)	< 10^–4^	90	79	64	95	88	41
Marker panel + PSA	0.94 (0.92–0.96)	< 10^–4^	90	82	65	96	89	43
Combined^d^	0.95 (0.93–0.97)	< 10^–4^	90	92	67	98	90	48
	Model II: (Benign + VLR/LR) vs (FIR + UIR + HR/VHR + mPC) (Marker number in model = 24)							
5 clinical factors^c^	0.82 (0.78–0.85)	< 10^–4^	90	42	65	78	75	26
Marker panel	0.85 (0.82–0.89)	< 10^–4^	90	63	74	85	82	40
Marker Panel + PSA	0.90 (0.88–0.93)	< 10^–4^	90	73	77	88	85	46
Combined^d^	0.92 (0.89–0.94)	< 10^–4^	90	75	77	89	86	47
	Model III: (Benign + VLR/LR + FIR) vs (UIR + HR/VHR + mPC) (Marker number in model = 26)							
5 clinical factors^c^	0.85 (0.82–0.88)	< 10^–4^	90	49	79	70	72	34
Marker panel	0.82 (0.78–0.85)	< 10^–4^	90	50	79	71	73	35
Marker Panel + PSA	0.90 (0.87–0.92)	< 10^–4^	90	67	84	79	80	47
Combined^d^	0.92 (0.90–0.94)	< 10^–4^	90	71	84	81	82	50
	Model GS: (Benign + GS < 7) vs (GS ≥ 7) (Marker number in model = 22)							
5 clinical factors^c^	0.78 (0.75–0.82)	< 10^–4^	90	30	65	67	67	19
Marker panel	0.80 (0.76–0.83)	< 10^–4^	90	46	74	73	73	29
Marker Panel + PSA	0.85 (0.81–0.87)	< 10^–4^	90	55	77	76	76	35
Combined^d^	0.87 (0.84–0.90)	< 10^–4^	90	56	78	77	77	36

P: *p* value for AUC (null hypothesis: AUC = 0.5)

*Sen* sensitivity, *Spe* specificity, *NPV* negative predictive value, *PPV* positive predictive value, *Bx* biopsy, *CI* confidence interval, *GS* Gleason score

^a^Sensitivity set at 90% for clinical relevance

^b^The percentage of biopsies avoided was calculated after the cohort was normalized to the original risk group composition of the entire cohort enrolled during the study period

^c^Five clinical risk factors; age, PSA value, family history of PC, previous negative biopsy for PC, abnormal DRE

^d^Metabolite marker panel plus 5 clinical factors. Table S3 (Additional file 1) shows similar statistics at 95% sensitivity

**Table 3:** Performance of the four predictive models (validation cohort, 90% sensitivity)

	AUC (95% CI)	P	Sen (%)^a^	Spe (%)	NPV (%)	PPV (%)	Accuracy (%)	Bx avoided (%)^b^
	Model I: Benign vs Cancer (Marker number in model = 26)							
5 clinical factors^c^	0.75 (0.70–0.80)	< 10^–4^	90	30	75	58	61	16
Marker panel	0.87 (0.83–0.91)	< 10^–4^	90	44	81	63	68	23
Marker panel + PSA	0.88 (0.84–0.92)	< 10^–4^	90	44	81	63	68	23
Combined^d^	0.89 (0.86–0.93)	< 10^–4^	90	57	85	69	74	30
	Model II: (Benign + VLR/LR) vs (FIR + UIR + HR/VHR + mPC) (Marker number in model = 24)							
5 clinical factors^c^	0.81 (0.76–0.86)	< 10^–4^	90	42	85	55	63	26
Marker panel	0.93 (0.90–0.95)	< 10^–4^	90	77	91	75	83	49
Marker Panel + PSA	0.94 (0.92–0.97)	< 10^–4^	90	81	91	78	85	51
Combined^d^	0.95 (0.93–0.97)	< 10^–4^	90	87	92	84	88	55
	Model III: (Benign + VLR/LR + FIR) vs (UIR + HR/VHR + mPC) (Marker number in model = 26)							
5 clinical factors^c^	0.84 (0.80–0.89)	< 10^–4^	90	51	91	50	65	36
Marker panel	0.88 (0.84–0.92)	< 10^–4^	90	70	93	63	78	49
Marker Panel + PSA	0.92 (0.92–0.96)	< 10^–4^	90	82	94	74	85	57
Combined^d^	0.93 (0.90–0.96)	< 10^–4^	90	84	94	76	86	59
	Model GS: (Benign + GS < 7) vs (GS ≥ 7) (Marker number in model = 22)							
5 clinical factors^c^	0.78 (0.72–0.83)	< 10^–4^	90	25	82	41	49	16
Marker panel	0.89 (0.86–0.93)	< 10^–4^	90	76	93	69	81	48
Marker Panel + PSA	0.91 (0.88–0.94)	< 10^–4^	90	79	94	71	83	50
Combined^d^	0.91 (0.88–0.94)	< 10^–4^	90	80	94	72	84	51

P: *p* value for AUC (null hypothesis: AUC = 0.5)

*Sen* sensitivity, *Spe* specificity, *NPV* negative predictive value, *PPV* positive predictive value, *Bx* biopsy, *CI* confidence interval, *GS* Gleason score

^a^Sensitivity set at 90% for clinical relevance

^b^The percent biopsy avoided was calculated after the cohort was normalized to the original risk group composition of the entire cohort enrolled during the study period

^c^Five clinical risk factors, including age, PSA value, family history of PC, previous negative biopsy for PC, abnormal DRE

^d^Metabolite marker panel plus 5 clinical factors. Table S3 (Additional file 1) shows similar statistics at 95% sensitivity

**Fig. 2:**
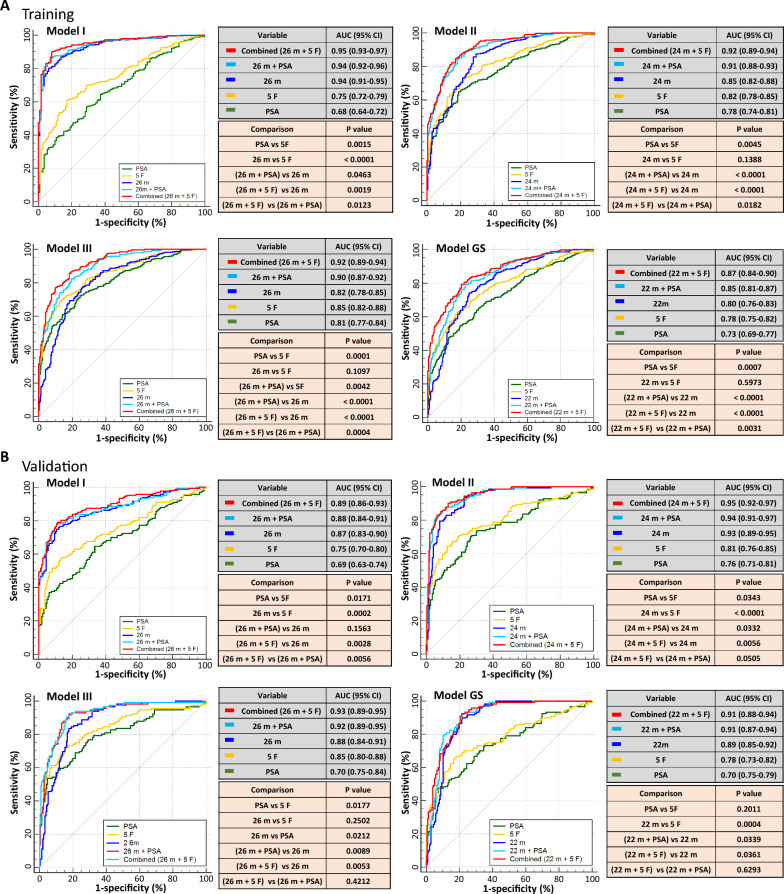
Area under the receiver operating characteristic curve analysis of the four predictive models for the training (**A)** and validation (**B**) cohorts. Model I was constructed to distinguish benign cases from all PC patients. Model II was constructed to distinguish isPC (benign + VLR/LR) from sPC (UIR + HR/VHR + mPC). Model III was constructed to distinguish isPC (benign + VLR/LR + FIR) from sPC (UIR + VHR/HR + mPC). Model GS was constructed to predict high-grade cancer (GS ≥ 7). PSA, prostate-specific antigen; isPC, insignificant prostate cancer; sPC, significant prostate cancer; VLR, very low risk; LR, low risk; FIR, favorable intermediate risk; UIR, unfavorable intermediate risk; HR, high risk; VHR, very high risk; mPC, metastatic prostate cancer; GS, Gleason score; AUC, area-under-the-curve. 26 m (in Models I and III): 26 metabolite markers, 24 m (in Model II): 24 metabolite markers, 22 m (in Model GS): 22 metabolite markers

**Fig. 3:**
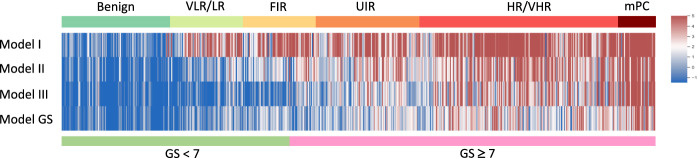
A heatmap of the four combined models. The heatmap was generated using patient logit values and the dichotomous method with the criterion corresponding to the Youden index J. It shows the probability of each patient belonging to a specific dichotomous end in four different models that combine distinct marker metabolites and clinical factors. Blue represents benign or isPC, while red represents PC or sPC. Darker color intensity reflects a higher probability of belonging to one end in the dichotomous models

As shown in Fig. 2 and Table 2 (training cohort), Model I distinguished benign cases from cancer cases with an AUC of 0.94, which was significantly higher than the AUCs for PSA (0.68, *p* < 0.0001) and the five clinical risk factors (0.75, *p* < 0.0001). The AUC of Model I was significantly improved by adding the PSA level (AUC = 0.94,* p* = 0.046) or clinical factors (AUC = 0.95, *p* = 0.0019). Model II had a higher AUC (0.85) than PSA (0.78, *p* = 0.0046), but a similar AUC to clinical factors (0.82, *p* = 0.139) for predicting sPC, reaching an AUC of 0.92 when it was combined with clinical factors. Model III had an AUC of 0.82, similar to that of PSA (0.81, *p* = 0.693), and clinical factors (0.85, *p* = 0.110). The combined Model III (with both markers and clinical factors) had a significantly improved AUC of 0.92. The AUC (0.80) of Model GS was lower than that of Models II and III and higher than that of PSA (0.73, *p* = 0.0133), but it did not differ from that of clinical factors (0.78, *p* = 0.597). The AUC of the combined GS model increased significantly to 0.87, which was lower than that of the combined Models II (0.92) or III (0.92). In addition, heatmaps based on logit values demonstrated the relative effectiveness of the four models in predicting PC and sPC (Fig. 3 and Additional file 1: Fig. S3). Furthermore, the heatmaps of the panel metabolites in the four models are presented in Additional file 1, Fig. S4.

### Validation

The four predictive models were successfully validated in the independent external cohort (N = 325) (Fig. 2 and Table 3), which had a risk group composition similar to the entire subject pool at enrollment (N = 1151) (Table 1). The results showed that these models did not overfit and were robust for predicting either PC (Model I) or sPC (Models II, III, and GS). Additional validation was conducted in two subgroups of men, namely, those aged ≥ 70 years and those with PSA levels less than 10 ng/ml, and the results showed that the models also performed well in the two subgroups, with AUCs similar to that of the entire validation cohort (Additional file 1: Tables S4 and S5).

### Avoidance of unnecessary biopsies

At 90% sensitivity, the marker panels in Models I, II, III, and GS could have avoided 41%, 40%, 35%, and 29% of unnecessary biopsies, respectively (Table 2). These percentages increased to 48%, 47%, 50%, and 36%, respectively, in the combined models. The corresponding statistics for the validation cohort are shown in Table 3 and Additional file 1: Table S3.

### Decision curve analysis

**Fig. 4:**
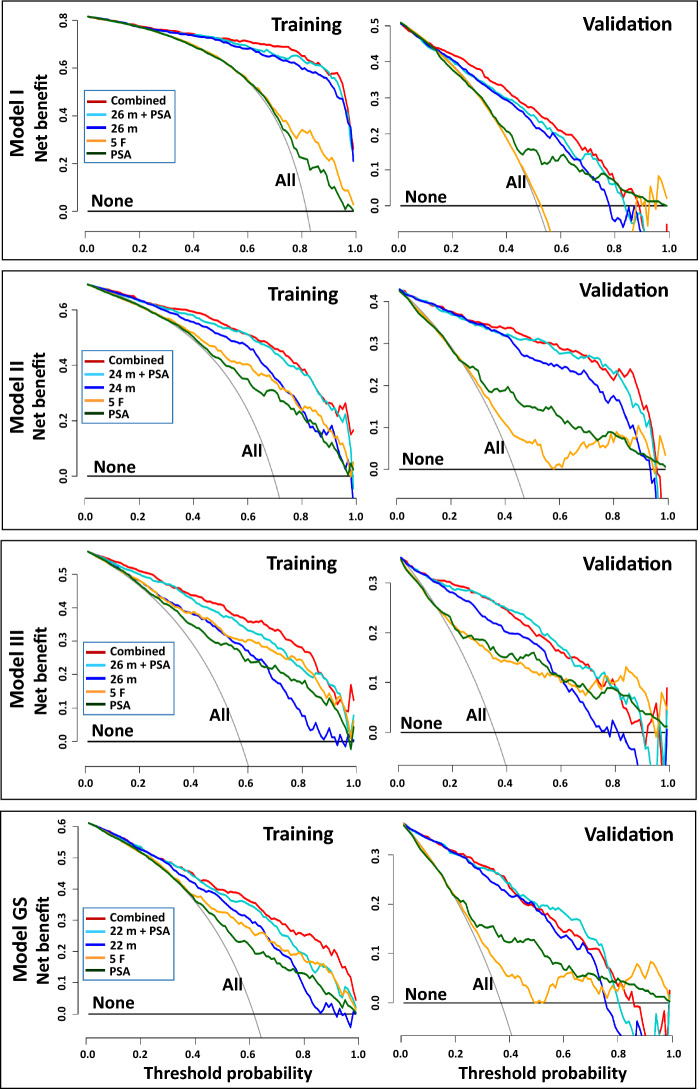
Decision curve analysis results of the four predictive models. None, biopsy-for-none; All, biopsy-for-all; 26 m (in Models I & III), 26 metabolite markers; 24 m (in Model II), 24 metabolite markers; 22 m (in Model GS), 22 metabolite markers. The net clinical benefit was greater in the validation cohort than in the training cohort in all four models

DCA showed that the combined Models II and III had greater clinical net benefit than the marker panels, clinical risk factors, or PSA alone (Fig. 4). However, the addition of PSA or clinical risk factors to the panel did not improve the net benefit of Model I. Notably, in Models II, III, and GS, DCA showed a significantly greater benefit in the validation cohort than in the training cohort, probably because the validation cohort had a proportion of benign cases (48.6%) more similar to those (52.0%) in the entire subject pool compared with the training cohort (18.2%) (Table 1). In contrast, the training cohort comprised 18.2% subjects with benign disease and 81.8% PC patients, resulting in a rightward shift of the 'biopsy-for-all' curve (indicating a higher threshold probability), thereby compressing the net benefit of the models. At a 10% threshold probability, the numbers of biopsies potentially avoided per 1,000 at-risk subjects in the training cohort were 50, 35, and 43 for Models II, III, and GS (marker panel alone), and 70, 148, and 33 for the combined models, respectively.

## Discussion

Metabolomics reveals functional information about the interactions of genes and the environment, unique from other omics approaches. Although progress has been made in urine metabolomics for PC biomarker research [38, 39, 46], a universally recognized biomarker/panel for predicting PC or sPC remains elusive. The urine metabolomics models presented in this study, especially the combined models, robustly predicted PC or sPC before biopsy in men at risk. Our urine-based tests have several advantages over others. First, we used the NCCN risk groupings instead of GS ≥ 7, which is used in most other popular tests [18, 21, 31, 47, 48], because sPC may not necessarily be a GS ≥ 7 disease and not only grading but also tumor staging may impact oncological outcomes. In contrast, small GS 3 + 4 tumors with low Gleason score 4 lesions may also be managed conservatively [4]. Second, our models were constructed to predict disease phenotypes rather than cancer potential, as reflected by the GS. The results showed that Models II and III outperformed Model GS, which also confirmed this advantage. In addition, according to the NCCN guidelines [4], FIR PC can be managed by active surveillance or watchful waiting, depending on life expectancy and tumor phenotype. In this study, we designed Models II and III for two different scenarios in which FIR PC was regarded as sPC and isPC, respectively [4]. This strategy allows flexible application by both physicians and men at risk.

In our study, the benign control group comprised men with an elevated risk of PC but a negative biopsy rather than healthy men without an elevated PSA level or men with benign prostatic hyperplasia (BPH) without any suspicion of PC. A limited number of previous studies reported urine tests that predicted PC with AUCs > 0.90 [49, 50]. One study demonstrated a high AUC (0.98) for differentiating PC patients from healthy controls but a low specificity (< 53%) for differentiating PC patients from those with BPH without elevated risk [49], which suggests that differences in urine metabolome profiles between PC patients and healthy controls are greater than those between PC and BPH patients. Similarly, differences in urine profiles between men with PC and benign controls at risk, as in our study, may be much more difficult to detect, but they are more clinically relevant. Therefore, previous studies [39, 51] that enrolled men at no risk as controls may not have addressed the real unmet need because men without risk of PC do not need to be evaluated for PC or sPC.

Previously published liquid biopsy models that adopted readily available clinical factors exhibited limited power in predicting high-grade PC (AUCs 0.61–0.77) [18, 20, 21]. Leyten et al. demonstrated that adding serum PSA to a urine 3-mRNA panel (*HOXC6*, *TDRD1*, and *DLX1*) increased the AUC from 0.77 to 0.81 [52]. Van Neste et al. combined urinary *HOXC6* and *DLX1* mRNA levels with several clinical factors, obtaining AUCs of 0.85–0.90 [31]. The results of these studies suggest that, although better than PSA alone, conventional clinical factors are limited in predicting high-grade PC, and including molecular markers may better stratify disease risks. Our results also showed that compared with clinical risk factors, metabolite markers, or PSA alone, the combined Models II, III, and GS showed improved AUCs (0.92, 0.92, and 0.87, respectively) for sPC prediction.

mpMRI has also been recommended to inform biopsy [23]. However, evidence has indicated a wide discrepancy among the findings of radiologists at the same center regarding the Prostate Imaging Reporting and Data System (PI-RADS) scores and cancer detection, with high-grade PC detection rates ranging from 40 to 80% for PI-RADS 5 lesions [53]. Such discrepancies could be even greater across institutions [54]. These data highlight the importance of objective tests or a combination of both. In our case, it may be inappropriate to combine the urine tests with mpMRI before biopsy. Because the AUCs of our models were sufficiently high, combining our tests and mpMRI, which would not be cost-effective, may not have been necessary. Therefore, we recommend a new strategy in which urine tests are conducted for men at risk and mpMRI should only be implemented in men with model-predicted PC or sPC, and then used to evaluate the necessity for a targeted biopsy, which may be more cost-effective because it would reduce the number of MRI evaluations in patients with benign disease. A previous study proposed a similar strategy in which mpMRI would be performed only in SelectMDx-positive men if quality mpMRI was not readily available [47].

Of note, the metabolite markers identified in our study may represent novel targets for PC research. Some markers, such as guanidinoacetic acid, 4-acetamidobutyric acid, pseudouridine, and monopalmitin, appeared repeatedly in three or four models. The first three markers increased, while the last one decreased in the PC or sPC. Guanidinoacetic acid, an arginine metabolite, is a precursor for the biosynthesis of creatine, which has been demonstrated in recent studies to promote tumor invasion and metastasis [55]. Whether the enriched guanidinoacetic acid in sPC acts as an oncometabolite to promote progression through creatine or other pathways remains to be investigated. 4-Acetamidobutyric acid is another arginine metabolite; its production requires monoamine oxidase, an actionable target in PC, the inhibition of which blocks the growth of castration-sensitive and castration-resistant cancers [56]. Pseudouridine is considered a potential biomarker for several cancers, including PC [57]. Monopalmitin, a monoglycerol ester of palmitic acid, has been reported to be decreased in the metabolome of lung cancer cells compared to normal epithelial cells [58]. In addition, the consistent prominence of monopalmitin and 1-stearoyl-rac-glycerol in our bubble plots suggested that these two specific metabolites held particular value in the context of our study. 1-Stearoylglycerol, a long-chain fatty alcohol, is formed as a product of lipid catabolism. Notably, men with elevated levels of serum 1-stearoylglycerol were reported to have a reduced likelihood of developing PC [59]. Further study of their roles in carcinogenesis may reveal crucial mechanisms and therapeutic targets in PC.

Our models may have a great translational impact for several reasons. First, they surpassed or equaled the performance of most other liquid biopsies [32–34, 38, 39, 46], including the widely used PCA3 [48] and SelectMDx [47] tests, as well as the most recent ones [60, 61], greatly improving the accuracy of predicting sPC before biopsy. Second, our data showed that unnecessary biopsies could be reduced, lessening the burden on patients and healthcare resources. Third, our DCA demonstrated a significant clinical benefit in Models II and III at lower threshold probabilities. Additionally, our models are noninvasive, eliminating the need for prostate massage or RNA handling, and offer enhanced clinical relevance through the NCCN risk groupings, while also being tailored to patients of different age groups compared to other popular models [38, 39, 46] in PC and sPC diagnoses. In the future, to optimize the effectiveness of our tests and maximize their impact, we will utilize targeted GC‒MS to accurately quantify metabolite markers. We will also assess the feasibility and prediction rate of using mpMRI to guide biopsy after positive results of our tests and compare it to other strategies. This novel strategy has the potential to change the landscape of PC management for at-risk men. Last, long-term utility analyses with diverse racial groups will determine whether our models are able to reduce unnecessary biopsies and overtreatment, without increasing the PC-specific mortality in the long run.

Finally, despite making headway, this study still had several limitations. First, because all subjects enrolled in our study were of Asian ethnicity, the generalizability of our results to other ethnic groups may be limited. However, our pioneering work may provide a foundation for future studies based on other ethnic groups. Second, our models apply to men at risk, but not to men at no risk, indicating that they may not be used to screen healthy men without risk. Third, most of our subjects did not receive prebiopsy MRI or postbiopsy molecular tests (e.g., Decipher^®^ Prostate Cancer Test). Therefore, we could not compare or combine our tests with mpMRI and other molecular tests.

## Conclusions

The models presented in this study, which combined urine metabolite markers and five clinical risk factors, predict NCCN-based sPC with very high accuracy. The two different sPC-predictive Models II and III may be applied to men with varied life expectancies. These novel urine tests may substantially address the unmet clinical need by effectively informing biopsy and avoiding approximately 40% of unnecessary biopsies, thus greatly modifying current clinical practice in the management of men with an elevated risk of PC.

### Supplementary Information

**Additional file 1:  Fig. S1**: Determination of marker numbers from the 30 top-ranking metabolites per the AIC principle for construction of the four predictive models. **Fig. S2**: Bubble plots for panel metabolites in the four models using the training cohort. **Fig. S3 **: Heatmaps of different markers/metabolites/panels in four combined models. **Fig. S4** Hierarchical maps of panel metabolites for the four predictive models using the validation cohort. **Table S1**: Metabolite marker panels and the respective chemical properties in the four predictive models. **Table S2**: Additional logistic regression parameters for marker panels in the training and validation cohorts. **Table S3**: Performance of the four predictive models (training and validation cohorts, at 95% of sensitivity). **Table S4**: Performance of the four predictive models in a subgroup with age more than or equal to 70 years (validation, 90% sensitivity). **Table S5**: Performance of the four predictive models in a subgroup with PSA levels less than 10 ng/ml (validation, 90% sensitivity).
