# Supplementary material for: Prediction of clinically significant prostate cancer through urine metabolomic signatures: A large-scale validated study
Source: J Transl Med. 2023 Oct 11;21:714. doi: 10.1186/s12967-023-04424-9 (PMC10566053; doi:10.1186/s12967-023-04424-9)
Supplement: Supplementary file 1 — Additional file 1: Fig. S1: Determination of marker numbers from the 30 top-ranking metabolites per the AIC principle for construction of the four predictive models. Fig. S2: Bubble plots for panel metabolites in the four models using the training cohort. Fig. S3 : Heatmaps of different markers/metabolites/panels in four combined models. Fig. S4 Hierarchical maps of panel metabolites for the four predictive models using the validation cohort. Table S1: Metabolite marker panels and the respective chemical properties in the four predictive models. Table S2: Additional logistic regression parameters for marker panels in the training and validation cohorts. Table S3: Performance of the four predictive models (training and validation cohorts, at 95% of sensitivity). Table S4: Performance of the four predictive models in a subgroup with age more than or equal to 70 years (validation, 90% sensitivity). Table S5: Performance of the four predictive models in a subgroup with PSA levels less than 10 ng/ml (validation, 90% sensitivity). [file 12967_2023_4424_MOESM1_ESM.docx]

**Additional files**

**Prediction of clinically significant prostate cancer using urine metabolomic signatures: A large-scale validated study**

H.-P. Huang, et al.

**Additional file 1:**

**Table of Contents**

Additional file Figures...........................................................................................................................2

*Figure S1*: Determination of marker numbers from the 30 top-ranking metabolites per the AIC principle for construction of the four predictive models.............................................2

*Figure S2*: Bubble plots for panel metabolites in four models using the training cohort…..……3

*Figure S3*: Heatmaps of different markers/metabolites/panels in four combined models…….....4

*Figure S4*: Hierarchical maps of panel metabolites for four predictive models........…...……….5

Supplementary Tables……………………………………………………………………………….....6

*Table S1*: Metabolite marker panels and the respective chemical properties in the four predictive models ...........................................................................................................................6

*Table S2*: Additional logistic regression parameters for marker panels in the training and validation cohorts………………………………………………………………….…11

*Table S3*: Metabolite marker panels and the respective chemical properties in the four predictive models .........................................................................................................................13

*Table S4*: Performance of the four predictive models in a subgroup with age more than or equal to 70 years (validation, 90% sensitivity) ….………………………………………....15

*Table S5*: Performance of the four predictive models in a subgroup with PSA level less than 10 ng/ml (validation, 90% sensitivity) ..….……………………………..........................16

**Additional file Figures**

**Fig. S1.**

**
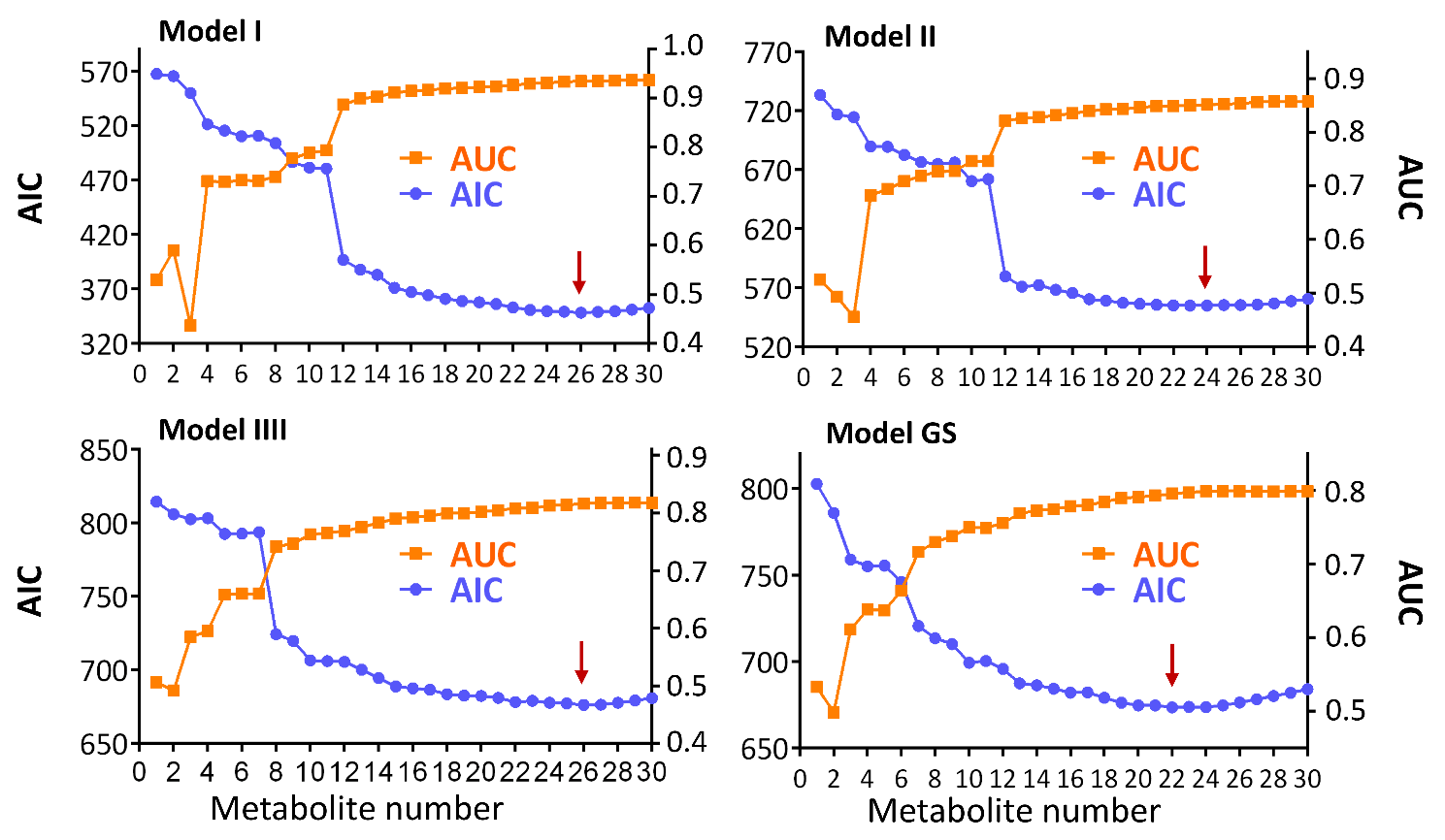
**

**Fig. S1** Determination of marker numbers from the 30 top-ranking metabolites per the AIC principle for construction of the four predictive models. Red arrows indicate AIC nadirs, and the optimal marker numbers needed for respective predictive models. Model I is used to predict all prostate cancer cases. Model II distinguishes isPC (benign+VLR/LR) from sPC (FIR+UIR+HR/VHR+mPC). Model III distinguishes isPC (benign+VLR/LR+FIR) from sPC (UIR+VHR/HR+mPC). Model GS is used to predict high-grade (GS ≥ 7) cancer. isPC: insignificant prostate cancer; sPC: significant prostate cancer; VLR: very low risk; LR: low risk; FIR: favorable intermediate risk; UIR: unfavorable intermediate risk, HR: high risk; VHR: very high risk; mPC: metastatic prostate cancer; GS: Gleason score; AIC: Akaike information criterion; AUC: area under the curve.

**Fig. S2**

**
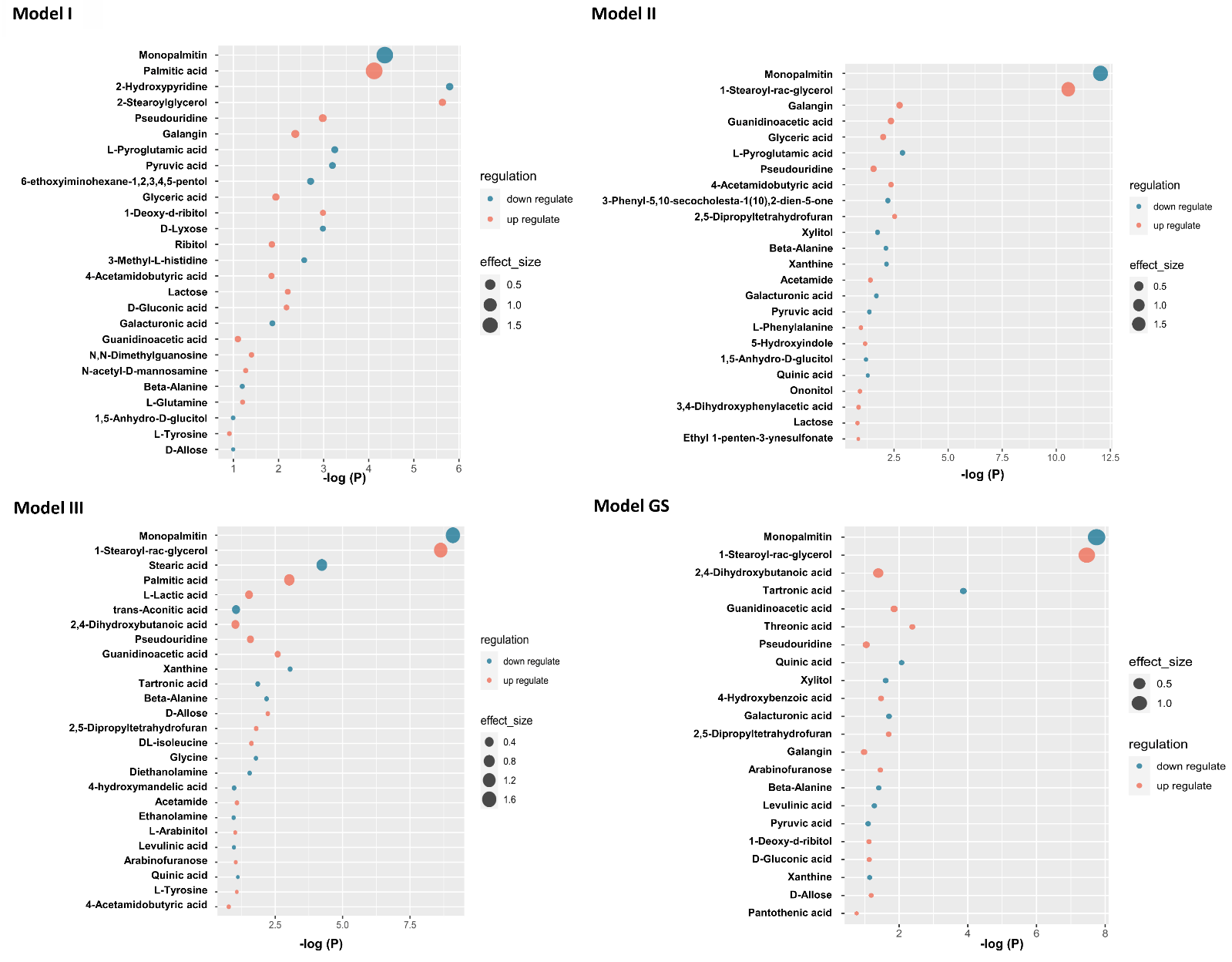
**

**Fig. S2** Bubble plots for panel metabolites in the four models using the training cohort. Bubble plots were created using the ggplot2 package in R. The x-axis term '-log (P)' represents the negative logarithm (base 10) of the *p* values derived from multivariable logistic regression for each model. The effect size is the absolute value of the natural logarithm of the odds ratio in logistic regression models. The ranking was determined according to the product of –log_10_ (P) and effect size. Tomato color: upregulated in cancer or sPC; green color: downregulated in cancer or sPC.

**Fig. S3**


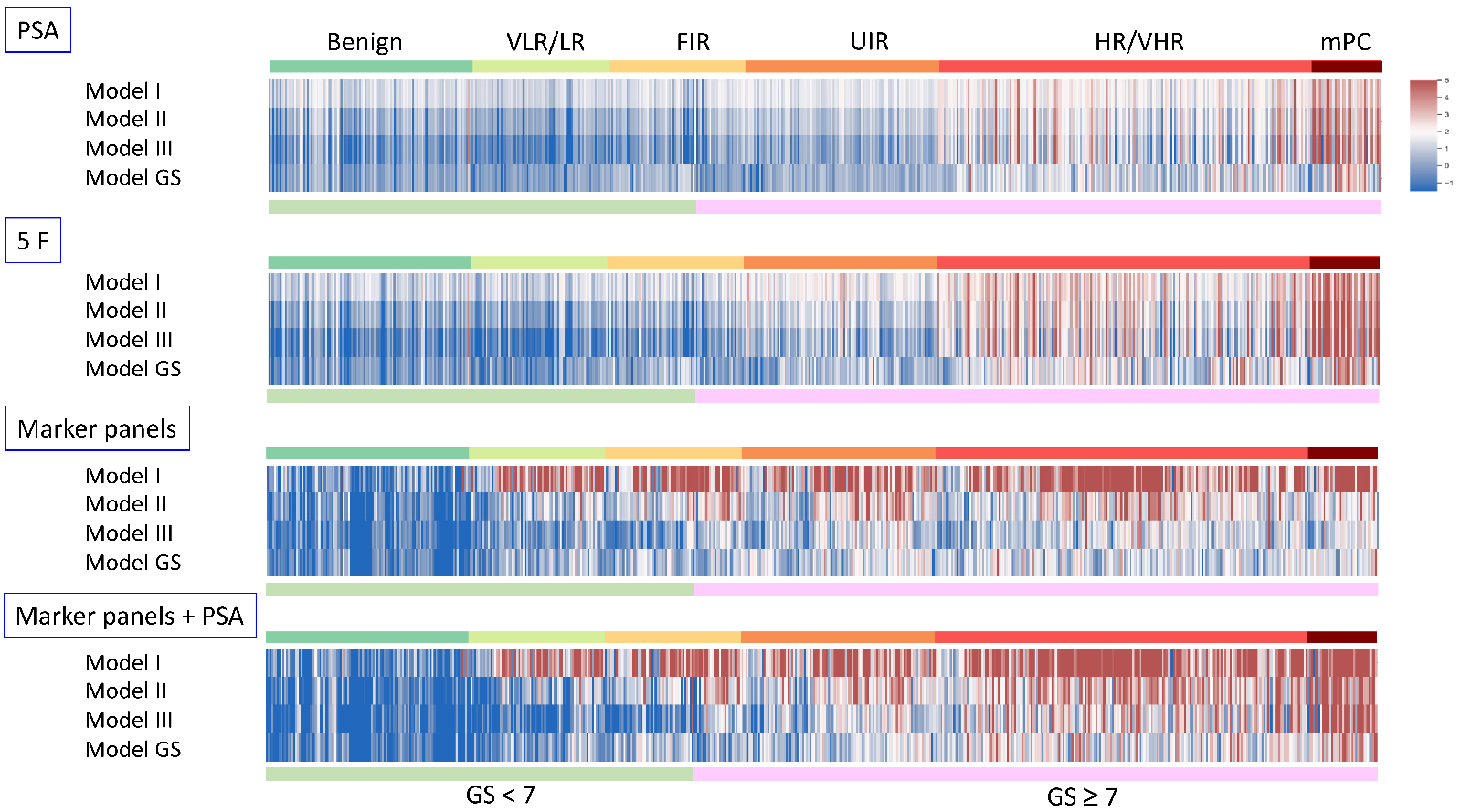


**Fig. S3** Comparison of heatmaps for different clinical factors and marker panels with and without PSA across four models using the training cohort. The heatmap, generated using the logit value of each patient and the dichotomous method based on Youden's index threshold, illustrates the predicted probabilities of each patient belonging to distinct subclasses determined by four different models. Darker color intensity reflects a higher likelihood of belonging to one end in the dichotomous model. 5 F: five clinical factors

**Fig. S4**

**
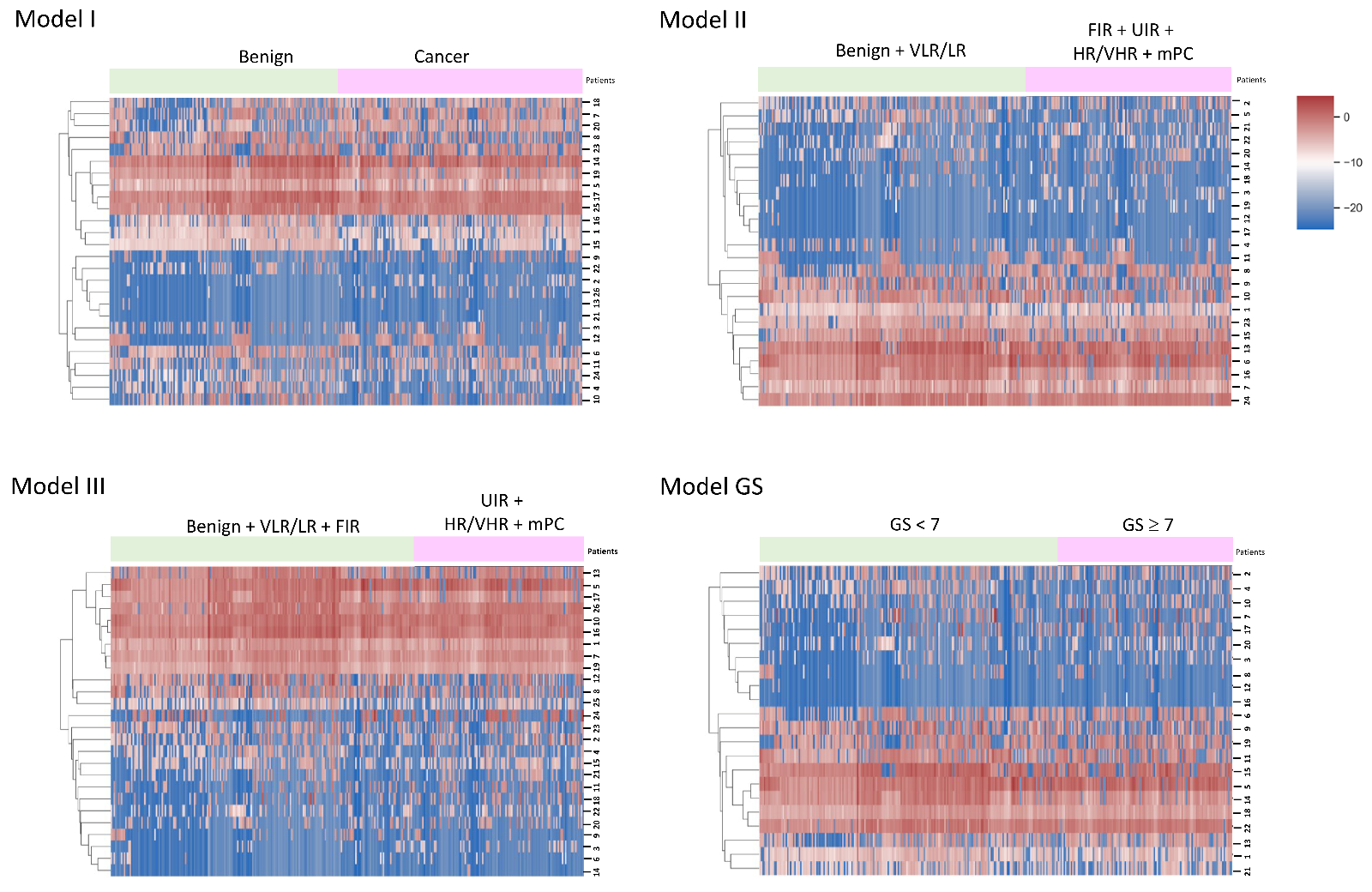
**

**Fig. S4** Hierarchical maps of panel metabolites for the four predictive models using the validation cohort. Panel metabolites were hierarchically clustered through the default “average” linkage method, which calculated the average Euclidean distance between all pairs of data points in different clusters. The order of the metabolite numbers is the same as those in Table 1.

**Supplementary Tables**

**Table S1. Metabolite marker panels and the respective chemical properties in the four predictive models**

| **Model I metabolite markers** | **Original form** | **CAS**  **Number** | **MW**  **g/mol** | **Molecular formula** | **Library found** | **Retention time (min)** | **Increased**  **in** |
| --- | --- | --- | --- | --- | --- | --- | --- |
| [1060] pyruvic acid [6.714] | Pyruvic acid | 127-17-3 | 88.06 | C3H4O3 | Fiehn | 6.60 | Benign |
| [18189] 4-acetamidobutyric acid 1 [12.863] | 4-Acetamidobutyric acid | 3025-96-5 | 145.16 | C6H11NO3 | Fiehn | 12.84 | Cancer |
| [219984] 1,5-anhydro-D-sorbitol [16.967] | 1,5-Anhydro-D-glucitol | 154-58-5 | 164.16 | C6H12O5 | Fiehn | 16.97 | Benign |
| [239] Beta- alanine 1 [12.044] | Beta-Alanine | 107-95-9 | 89.09 | C3H7NO2 | Fiehn | 12.04 | Benign |
| [439194] glyceric acid [10.735] | Glyceric acid | 473-81-4 | 106.08 | C3H6O4 | Fiehn | 10.63 | Cancer |
| [439240] D-lyxose 2 [14.889] | D-Lyxose | 1114-34-7 | 150.13 | C5H10O5 | Fiehn | 14.81 | Benign |
| [445929] galacturonic acid 2 [18.105] | Galacturonic acid | 685-73-4 | 194.14 | C6H10O7 | Fiehn | 17.96 | Benign |
| [448388] D-allose 1 [17.278] | D-Allose | 2595-97-3 | 180.16 | C6H12O6 | Fiehn | 19.36 | Benign |
| [6057] L-tyrosine 2 [17.856] | L-Tyrosine | 60-18-4 | 181.19 | C9H11NO3 | Fiehn | 17.78 | Cancer |
| [64969] 3-methyl-L-histidine [16.423] | 3-Methyl-L-histidine | 368-16-1 | 169.18 | C7H11N3O2 | Fiehn | 16.42 | Benign |
| [738] L-glutamine 2 [14.083] | L-Glutamine | 56-85-9 | 146.14 | C5H10N2O3 | Fiehn | 14.08 | Cancer |
| [7405] L-pyroglutamic acid [13.218] | L-Pyroglutamic acid | 98-79-3 | 129.11 | C5H7NO3 | Fiehn | 13.14 | Benign |
| [763] guanidinoacetic acid 2 [14.751] | Guanidinoacetic acid | 352-97-6 | 117.11 | C3H7N3O2 | Fiehn | 14.70 | Cancer |
| [84571] lactose 1 [24.386] | Lactose | 63-42-3 | 342.3 | C12H22O11 | Fiehn | 24.39 | Cancer |
| [8871] 2-hydroxypyridine [6.519] | 2-Hydroxypyridine | 142-08-05 | 95.1 | C5H5NO | Fiehn | 6.52 | Benign |
| [899] N-acetyl-D-mannosamine 1 [19.177] | N-acetyl-D-mannosamine | 7772-94-3 | 221.21 | C8H15NO6 | Fiehn | 19.18 | Cancer |
| [985] palmitic acid [18.846] | Palmitic acid | 57-10-3 | 256.4 | C16H32O2 | Fiehn | 18.85 | Cancer |
| 1-Deoxypentitol, 4TMS derivative | 1-Deoxy-d-ribitol | 13046-76-9 | 136.15 | C5H12O4 | NIST | 13.30 | Cancer |
| 1-Monopalmitin, 2TMS derivative | Monopalmitin | 542-44-9 | 330.5 | C19H38O4 | NIST | 23.47 | Benign |
| 2-Monostearin, 2TMS derivative | 2-Stearoylglycerol | 621-61-4 | 358.56 | C21H42O4 | NIST | 24.65 | Cancer |
| 2-phenyl-3,5,7-tris(trimethylsilyloxy)-1-benzopyran-4-one | Galangin | 548-83-4 | 270.24 | C15H10O5 | NIST | 26.09 | Cancer |
| D-Allose, pentakis(trimethylsilyl) ether, ethyloxime (isomer 2) | 6-ethoxyiminohexane-1,2,3,4,5-pentol | 2058302-87-5 | 223.22 | C8H17NO6 | NIST | 15.90 | Benign |
| D-Gluconic acid, 6TMS derivative | D-Gluconic acid | 526-95-4 | 196.16 | C6H12O7 | NIST | 18.21 | Cancer |
| Guanosine, N,N-dimethyl-1-(trimethylsilyl)-2',3',5'-tris-O-(trimethylsilyl)- | N,N-Dimethylguanosine | 2140-67-2 | 311.29 | C12H17N5O5 | NIST | 25.14 | Cancer |
| Pseudo uridine penta-tms | Pseudouridine | 1445-07-04 | 244.2 | C9H12N2O6 | NIST | 21.40 | Cancer |
| Ribitol TMS | Ribitol | 488-81-3 | 152.15 | C5H12O5 | NIST | 15.96 | Cancer |
|  | | | | | | | |
| **Model II metabolite markers** | **Original form** | **CAS**  **Number** | **MW**  **g/mol** | **Molecular formula** | **Library found** | **Retention time (min)** | **Increased**  **in** |
| [1060] pyruvic acid [6.714] | Pyruvic acid | 127-17-3 | 88.06 | C3H4O3 | Fiehn | 6.60 | Benign + isPC |
| [1188] xanthine [18.574] | Xanthine | 69-89-6 | 152.11 | C5H4N4O2 | Fiehn | 18.57 | Benign + isPC |
| [18189] 4-acetamidobutyric acid 1 [12.863] | 4-Acetamidobutyric acid | 3025-96-5 | 145.16 | C6H11NO3 | Fiehn | 12.84 | sPC |
| [219984] 1,5-anhydro-D-sorbitol [16.967] | 1,5-Anhydro-D-glucitol | 154-58-5 | 164.16 | C6H12O5 | Fiehn | 16.97 | Benign + isPC |
| [239] Beta- alanine 1 [12.044] | Beta-Alanine | 107-95-9 | 89.09 | C3H7NO2 | Fiehn | 12.04 | Benign + isPC |
| [24699] 1-stearoyl-rac-glycerol [24.913] | 1-Stearoyl-rac-glycerol | 123-94-4 | 358.6 | C21H42O4 | Fiehn | 24.91 | Benign + isPC |
| [439194] glyceric acid [10.735] | Glyceric acid | 473-81-4 | 106.08 | C3H6O4 | Fiehn | 10.63 | sPC |
| [445929] galacturonic acid 2 [18.105] | Galacturonic acid | 685-73-4 | 194.14 | C6H10O7 | Fiehn | 17.96 | Benign + isPC |
| [6508] quinic acid [17.076] | Quinic acid | 77-95-2 | 192.17 | C7H12O6 | Fiehn | 17.08 | Benign + isPC |
| [6912] xylitol [15.376] | Xylitol | 87-99-0 | 152.15 | C5H12O5 | Fiehn | 15.69 | Benign + isPC |
| [7405] L-pyroglutamic acid [13.218] | L-Pyroglutamic acid | 98-79-3 | 129.11 | C5H7NO3 | Fiehn | 13.14 | Benign + isPC |
| [763] guanidinoacetic acid 2 [14.751] | Guanidinoacetic acid | 352-97-6 | 117.11 | C3H7N3O2 | Fiehn | 14.70 | sPC |
| [84571] lactose 1 [24.386] | Lactose | 63-42-3 | 342.3 | C12H22O11 | Fiehn | 24.39 | sPC |
| Ononitol TMS | Ononitol | 6090-97-7 | 194.18 | C7H14O6 | NIST | 23.80 | sPC |
| 1H-Indole, 1-(trimethylsilyl)-5-[(trimethylsilyl)oxy]- | 5-Hydroxyindole | 1953-54-4 | 133.15 | C8H7NO | NIST | 15.35 | sPC |
| 1-Monopalmitin, 2TMS derivative | Monopalmitin | 542-44-9 | 330.5 | C19H38O4 | NIST | 23.47 | Benign + isPC |
| 2-phenyl-3,5,7-tris(trimethylsilyloxy)-1-benzopyran-4-one | Galangin | 548-83-4 | 270.24 | C15H10O5 | NIST | 26.09 | sPC |
| 3,4-Dihydroxyphenylacetic Acid, 3TMS derivative | 3,4-Dihydroxyphenylacetic acid | 102-32-9 | 168.15 | C8H8O4 | NIST | 16.65 | sPC |
| 3-Phenyl-5,10-secocholesta-1(10),2-dien-5-one | 3-Phenyl-5,10-secocholesta-1(10),2-dien-5-one | --* | 460.74 | C33H48O | NIST | 23.31 | Benign + isPC |
| Ethanimidic acid, N-(trimethylsilyl)-, trimethylsilyl ester | Acetamide | 60-35-5 | 59.07 | C2H5NO | NIST | 8.56 | sPC |
| Ethyl (E)-1-penten-3-ynesulfonate | Ethyl 1-penten-3-ynesulfonate | 171816-65-2 | 174.22 | C7H10O3S | NIST | 13.86 | sPC |
| Furan, tetrahydro-2,5-dipropyl- | 2,5-Dipropyltetrahydrofuran | 4457-62-9 | 156.26 | C10H20O | NIST | 7.33 | sPC |
| L-Phenylalanine, 2TMS derivative | L-Phenylalanine | 63-91-2 | 165.19 | C9H11NO2 | NIST | 14.41 | sPC |
| Pseudo uridine penta-tms | Pseudouridine | 1445-07-04 | 244.2 | C9H12N2O6 | NIST | 21.40 | sPC |
|  | | | | | | | |
| **Model III metabolite markers** | **Original form** | **CAS**  **Number** | **MW**  **g/mol** | **Molecular formula** | **NIST**  **library** | **Retention time (min)** | **Increased**  **in** |
| [107689] L-(+) lactic acid [6.851] | L-Lactic acid | 79-33-4 | 90.08 | C3H6O3 | Fiehn | 6.84 | sPC |
| [1188] xanthine [18.574] | Xanthine | 69-89-6 | 152.11 | C5H4N4O2 | Fiehn | 18.57 | Benign + isPC |
| [18189] 4-acetamidobutyric acid 1 [12.863] | 4-Acetamidobutyric acid | 3025-96-5 | 145.16 | C6H11NO3 | Fiehn | 12.84 | sPC |
| [239] Beta- alanine 1 [12.044] | Beta-Alanine | 107-95-9 | 89.09 | C3H7NO2 | Fiehn | 12.04 | Benign + isPC |
| [24699] 1-stearoyl-rac-glycerol [24.913] | 1-Stearoyl-rac-glycerol | 123-94-4 | 358.60 | C21H42O4 | Fiehn | 24.91 | Benign + isPC |
| [328] DL-4-hydroxymandelic acid [16.126] | 4-hydroxymandelic acid | 1198-84-1 | 168.15 | C8H8O4 | Fiehn | 16.12 | Benign + isPC |
| [444212] trans-aconitic acid [15.842] | trans-Aconitic acid | 4023-65-8 | 174.11 | C6H6O6 | Fiehn | 15.76 | sPC |
| [448388] D-allose 1 [17.278] | D-Allose | 2595-97-3 | 180.16 | C6H12O6 | Fiehn | 19.36 | Benign + isPC |
| [45] tartronic acid [11.523] | Tartronic acid | 80-69-3 | 120.06 | C3H4O5 | Fiehn | 11.52 | Benign + isPC |
| [5281] stearic acid [20.675] | Stearic acid | 57-11-4 | 284.48 | C18H36O2 | Fiehn | 20.67 | Benign + isPC |
| [6057] L-tyrosine 2 [17.856] | L-Tyrosine | 60-18-4 | 181.19 | C9H11NO3 | Fiehn | 17.78 | sPC |
| [6508] quinic acid [17.076] | Quinic acid | 77-95-2 | 192.17 | C7H12O6 | Fiehn | 17.08 | Benign + isPC |
| [700] ethanolamine [9.879] | Ethanolamine | 141-43-5 | 61.08 | C2H7NO | Fiehn | 9.88 | Benign + isPC |
| [763] guanidinoacetic acid 2 [14.751] | Guanidinoacetic acid | 352-97-6 | 117.11 | C3H7N3O2 | Fiehn | 14.70 | sPC |
| [791] DL-isoleucine 2 [10.225] | DL-isoleucine | 443-79-8 | 131.17 | C6H13NO2 | Fiehn | 10.23 | sPC |
| [985] palmitic acid [18.846] | Palmitic acid | 57-10-3 | 256.40 | C16H32O2 | Fiehn | 18.85 | sPC |
| 1-Monopalmitin, 2TMS derivative | Monopalmitin | 542-44-9 | 330.5 | C19H38O4 | NIST | 23.47 | Benign + isPC |
| Arabinofuranose, 1,2,3,5-tetrakis-O-(trimethylsilyl)- | Arabinofuranose | 13221-22-2 | 150.13 | C5H10O5 | NIST | 18.86 | sPC |
| Butanoic acid, 2,4-bis[(trimethylsilyl)oxy]-, trimethylsilyl ester | 2,4-Dihydroxybutanoic acid | 1518-62-3 | 120.10 | C4H8O4 | NIST | 11.73 | sPC |
| Diethanolamine, 3TMS derivative | Diethanolamine | 111-42-2 | 105.14 | C4H11NO2 | NIST | 11.86 | Benign + isPC |
| Ethanimidic acid, N-(trimethylsilyl)-, trimethylsilyl ester | Acetamide | 60-35-5 | 59.07 | C2H5NO | NIST | 8.56 | sPC |
| Furan, tetrahydro-2,5-dipropyl- | 2,5-Dipropyltetrahydrofuran | 4457-62-9 | 156.26 | C10H20O | NIST | 7.33 | sPC |
| Glycine, di-TMS | Glycine | 56-40-6 | 75.07 | C2H5NO2 | NIST | 7.76 | Benign + isPC |
| L-(-)-Arabitol, 5TMS derivative | L-Arabinitol | 7643-75-6 | 152.15 | C5H12O5 | NIST | 15.44 | sPC |
| Pentenoic acid, 4-[(trimethylsilyl)oxy]-, trimethylsilyl ester | Levulinic acid | 123-76-2 | 116.12 | C5H8O3 | NIST | 7.73 | Benign + isPC |
| Pseudo uridine penta-tms | Pseudouridine | 1445-07-04 | 244.20 | C9H12N2O6 | NIST | 21.40 | sPC |
|  | | | | | | | |
| **Model GS metabolite markers** | **Original form** | **CAS**  **Number** | **MW**  **g/mol** | **Molecular formula** | **Library found** | **Retention time (min)** | **Increased**  **in** |
| [1060] pyruvic acid [6.714] | Pyruvic acid | 127-17-3 | 88.06 | C3H4O3 | Fiehn | 6.60 | Benign + GS < 7 |
| [1188] xanthine [18.574] | Xanthine | 69-89-6 | 152.11 | C5H4N4O2 | Fiehn | 18.57 | Benign + GS < 7 |
| [135] 4-hydroxybenzoic acid [14.505] | 4-Hydroxybenzoic acid | 99-96-7 | 138.12 | C7H6O3 | Fiehn | 14.51 | GS ≥ 7 |
| [239] Beta- alanine 1 [12.044] | Beta-Alanine | 107-95-9 | 89.09 | C3H7NO2 | Fiehn | 12.04 | Benign + GS < 7 |
| [24699] 1-stearoyl-rac-glycerol [24.913] | 1-Stearoyl-rac-glycerol | 123-94-4 | 358.60 | C21H42O4 | Fiehn | 24.91 | Benign + GS < 7 |
| [445929] galacturonic acid 2 [18.105] | Galacturonic acid | 685-73-4 | 194.14 | C6H10O7 | Fiehn | 17.96 | Benign + GS < 7 |
| [448388] D-allose 2 [17.521] | D-Allose | 2595-97-3 | 180.156 | C6H12O6 | Fiehn | 19.36 | GS ≥ 7 |
| [45] tartronic acid [11.523] | Tartronic acid | 80-69-3 | 120.06 | C3H4O5 | Fiehn | 11.52 | Benign + GS < 7 |
| [6508] quinic acid [17.076] | Quinic acid | 77-95-2 | 192.17 | C7H12O6 | Fiehn | 17.08 | Benign + GS < 7 |
| [6613] pantothenic acid 2 [18.371] | Pantothenic acid | 79-83-4 | 219.23 | C9H17NO5 | Fiehn | 18.37 | GS ≥ 7 |
| [6912] xylitol [15.376] | Xylitol | 87-99-0 | 152.15 | C5H12O5 | Fiehn | 15.69 | Benign + GS < 7 |
| [763] guanidinoacetic acid 2 [14.751] | Guanidinoacetic acid | 352-97-6 | 117.108 | C3H7N3O2 | Fiehn | 14.70 | GS ≥ 7 |
| 1-Deoxypentitol, 4TMS derivative | 1-Deoxy-d-ribitol | 13046-76-9 | 136.15 | C5H12O4 | NIST | 13.30 | GS ≥ 7 |
| 1-Monopalmitin, 2TMS derivative | Monopalmitin | 542-44-9 | 330.5 | C19H38O4 | NIST | 23.47 | Benign + GS < 7 |
| 2,3,4-Trihydroxybutyric acid tetrakis(trimethylsilyl) deriv., (, (R*,R*)-) | Threonic acid | 3909/12/4 | 136.1 | C4H8O5 | NIST | 13.44 | GS ≥ 7 |
| 2-phenyl-3,5,7-tris(trimethylsilyloxy)-1-benzopyran-4-one | Galangin | 548-83-4 | 270.24 | C15H10O5 | NIST | 26.09 | GS ≥ 7 |
| Arabinofuranose, 1,2,3,5-tetrakis-O-(trimethylsilyl)- | Arabinofuranose | 13221-22-2 | 150.13 | C5H10O5 | NIST | 18.86 | GS ≥ 7 |
| Butanoic acid, 2,4-bis[(trimethylsilyl)oxy]-, trimethylsilyl ester | 2,4-Dihydroxybutanoic acid | 1518-62-3 | 120.10 | C4H8O4 | NIST | 11.73 | GS ≥ 7 |
| D-Gluconic acid, 6TMS derivative | D-Gluconic acid | 526-95-4 | 196.16 | C6H12O7 | NIST | 18.21 | GS ≥ 7 |
| Furan, tetrahydro-2,5-dipropyl- | 2,5-Dipropyltetrahydrofuran | 4457-62-9 | 156.26 | C10H20O | NIST | 7.33 | GS ≥ 7 |
| Pentenoic acid, 4-[(trimethylsilyl)oxy]-, trimethylsilyl ester | Levulinic acid | 123-76-2 | 116.12 | C5H8O3 | NIST | 7.73 | Benign + GS < 7 |
| Pseudo uridine penta-tms | Pseudouridine | 1445-07-04 | 244.2 | C9H12N2O6 | NIST | 21.40 | GS ≥ 7 |

CAS number: Chemical Abstracts Service Registry Number; NIST: National Institute of Standards and Technology; isPC: insignificant prostate cancer; sPC: significant prostate cancer; VLR: very low risk; LR: low risk; FIR: favourable intermediate risk; UIR: unfavourable intermediate risk; HR: high risk; VHR: very high risk; mPC: metastatic prostate cancer; GS, Gleason score; * no CAS No. is available, but its PubChem Compound ID number is 14928863.

**Table S2.** Additional logistic regression parameters for marker panels in the training and validation cohorts

| **Training Cohort** | | | | | | |
| --- | --- | --- | --- | --- | --- | --- |
| **Model I: Benign vs Cancer** (Marker number in model=26) | | | | | | |
|  | Youden Index J | Cut-off Sen. (%) | Cut-off Spe. (%) | Cox & Snell R^2^ | Nagelkerke R^2^ | P of Hosmer & Lemeshow test |
| 5 clinical factors^†^ | 0.44 | 61 | 83 | 0.11 | 0.19 | 0.106 |
| Marker panel | 0.74 | 80 | 94 | 0.37 | 0.60 | 0.942 |
| Marker panel + PSA | 0.78 | 87 | 92 | 0.39 | 0.63 | 0.846 |
| Combined^#^ | 0.82 | 90 | 92 | 0.41 | 0.66 | 0.491 |
| **Model II: (Benign + VLR/LR) vs (FIR + UIR + HR/VHR + mPC)** (Marker number in model=24) | | | | | | |
|  | Youden Index J | Cut-off Sen. (%) | Cut-off Spe. (%) | Cox & Snell R^2^ | Nagelkerke R^2^ | P of Hosmer & Lemeshow test |
| 5 clinical factors^†^ | 0.51 | 73 | 78 | 0.24 | 0.34 | 0.779 |
| Marker panel | 0.58 | 87 | 71 | 0.32 | 0.46 | 0.044 |
| Marker panel + PSA | 0.66 | 89 | 77 | 0.41 | 0.58 | 0.911 |
| Combined^#^ | 0.68 | 82 | 85 | 0.44 | 0.62 | 0.308 |
| **Model III: (Benign + VLR/LR + FIR) vs (UIR + HR/VHR + mPC)** (Marker number in model=26) | | | | | | |
|  | Youden Index J | Cut-off Sen. (%) | Cut-off Spe. (%) | Cox & Snell R^2^ | Nagelkerke R^2^ | P of Hosmer & Lemeshow test |
| 5 clinical factors^†^ | 0.56 | 70 | 86 | 0.35 | 0.47 | 0.259 |
| Marker panel | 0.51 | 77 | 74 | 0.28 | 0.38 | 0.462 |
| Marker panel + PSA | 0.63 | 82 | 81 | 0.43 | 0.58 | 0.331 |
| Combined^#^ | 0.67 | 86 | 82 | 0.48 | 0.65 | 0.528 |
| **Model GS: (Benign + GS < 7) vs GS** ≥ **7** (Marker number in model=22) | | | | | | |
|  | Youden Index J | Cut-off Sen. (%) | Cut-off Spe. (%) | Cox & Snell R^2^ | Nagelkerke R^2^ | P of Hosmer & Lemeshow test |
| 5 clinical factors^†^ | 0.45 | 63 | 82 | 0.23 | 0.31 | 0.292 |
| Marker panel | 0.49 | 74 | 75 | 0.25 | 0.34 | 0.393 |
| Marker panel + PSA | 0.56 | 79 | 77 | 0.33 | 0.45 | 0291 |
| Combined^#^ | 0.59 | 79 | 80 | 0.37 | 0.50 | 0.418 |
| **Validation cohort** | | | | | | |
| **Model I: Benign vs Cancer** (Marker number in model=26) | | | | | | |
|  | Youden Index J | Cut-off Sen. (%) | Cut-off Spe. (%) | Cox & Snell R^2^ | Nagelkerke R^2^ | P of Hosmer & Lemeshow test |
| 5 clinical factors^†^ | 0.44 | 57 | 87 | 0.19 | 0.26 | 0.016 |
| Marker panel | 0.64 | 75 | 89 | 0.39 | 0.52 | 0.189 |
| Marker panel + PSA | 0.68 | 78 | 89 | 0.41 | 0.55 | 0.064 |
| Combined^#^ | 0.68 | 78 | 89 | 0.44 | 0.58 | 0.487 |
| **Model II: (Benign + VLR and LR) vs (FIR + UIR + HR and VHR + mPC)** (Marker number in model=24) | | | | | | |
|  | Youden Index J | Cut-off Sen. (%) | Cut-off Spe. (%) | Cox & Snell R^2^ | Nagelkerke R^2^ | P of Hosmer & Lemeshow test |
| 5 clinical factors^†^ | 0.52 | 65 | 88 | 0.26 | 0.36 | 0.0017 |
| Marker panel | 0.71 | 79 | 92 | 0.49 | 0.65 | 0.003 |
| Marker panel + PSA | 0.77 | 88 | 89 | 0.53 | 0.71 | 0.021 |
| Combined^#^ | 0.77 | 90 | 87 | 0.55 | 0.73 | 0.545 |
| **Model III: (Benign + VLR and LR + FIR) vs (UIR + HR and VHR + mPC)** (Marker number in model=26) | | | | | | |
|  | Youden Index J | Cut-off Sen. (%) | Cut-off Spe. (%) | Cox & Snell R^2^ | Nagelkerke R^2^ | P of Hosmer & Lemeshow test |
| 5 clinical factors^†^ | 0.56 | 73 | 84 | 0.31 | 0.42 | 0.0253 |
| Marker panel | 0.66 | 84 | 82 | 0.38 | 0.52 | 0.332 |
| Marker panel + PSA | 0.75 | 92 | 82 | 0.46 | 0.64 | 0.511 |
| Combined^#^ | 0.74 | 91 | 84 | 0.48 | 0.65 | 0.224 |
| **Model GS: (Benign + GS < 7) vs GS** ≥ **7** (Marker number in model=22) | | | | | | |
|  | Youden Index J | Cut-off Sen. (%) | Cut-off Spe. (%) | Cox & Snell R^2^ | Nagelkerke R^2^ | P of Hosmer & Lemeshow test |
| 5 clinical factors^†^ | 0.49 | 66 | 83 | 0.24 | 0.32 | 0.165 |
| Marker panel | 0.69 | 90 | 79 | 0.41 | 0.56 | 0.116 |
| Marker panel + PSA | 0.70 | 92 | 79 | 0.45 | 0.61 | 0.293 |
| Combined^#^ | 0.71 | 93 | 79 | 0.45 | 0.61 | 0.386 |

^†^: 5 clinical risk factors; ^#^: metabolite marker panel plus 5 clinical risk factors; Sens: sensitivity; Spe: specificity.

**Table S3. Performance of the four predictive models (training and validation cohorts, at 95% of sensitivity)**

| **Training cohort** | |  | |  | |  | |  |  |  |  |  |
| --- | --- | --- | --- | --- | --- | --- | --- | --- | --- | --- | --- | --- |
|  | | AUC (95% CI) | | P | | Sen (%)* | | Spe (%) | NPV (%) | PPV (%) | Accuracy (%) | Bx avoided (%)^§^ |
|  | **Model I: Benign vs Cancer** (Marker number in model=26) | | | | | | | | | | | |
| 5 clinical factors^†^ | | | 0.75 (0.71–0.80) | <10^-4^ | | 95 | 18 | | 45 | 84 | 81 | 9 |
| Marker panel | | | 0.94 (0.91–0.96) | <10^-4^ | | 95 | 64 | | 75 | 92 | 89 | 33 |
| Marker panel + PSA | | | 0.94 (0.92–0.96) | <10^-4^ | | 95 | 69 | | 76 | 93 | 90 | 36 |
| Combined^#^ | | | 0.95 (0.93–0.97) | <10^-4^ | | 95 | 73 | | 77 | 94 | 91 | 38 |
|  | **Model II: (Benign + VLR/LR) vs (FIR + UIR + HR/VHR + mPC)** (Marker number in model=24) | | | | | | | | | | | |
| 5 clinical factors^†^ | | | 0.82 (0.78–0.85) | <10^-4^ | | 95 | 28 | | 72 | 75 | 75 | 18 |
| Marker panel | | | 0.85 (0.82–0.89) | <10^-4^ | | 95 | 48 | | 81 | 81 | 81 | 30 |
| Marker Panel + PSA | | | 0.90 (0.88–0.93) | <10^-4^ | | 95 | 56 | | 84 | 83 | 83 | 35 |
| Combined^#^ | | | 0.92 (0.89–0.94) | <10^-4^ | | 95 | 68 | | 86 | 87 | 87 | 43 |
|  | **Model III: (Benign + VLR/LR + FIR) vs (UIR + HR/VHR + mPC)** (Marker number in model=26) | | | | | | | | | | | |
| 5 clinical factors^†^ | | | 0.85 (0.82–0.88) | <10^-4^ | | 95 | 36 | | 84 | 66 | 70 | 25 |
| Marker panel | | | 0.82 (0.78–0.85) | <10^-4^ | | 95 | 34 | | 84 | 66 | 69 | 24 |
| Marker Panel + PSA | | | 0.90 (0.87–0.92) | <10^-4^ | | 95 | 61 | | 90 | 76 | 80 | 43 |
| Combined^#^ | | | 0.92 (0.90-0.94) | <10^-4^ | | 95 | 60 | | 90 | 76 | 80 | 42 |
|  | **Model GS: (Benign + GS <7) vs (GS** ≥ **7)** (Marker number in model=22) | | | | | | | | | | |  |
| 5 clinical factors^†^ | | | 0.78 (0.75–0.82) | <10^-4^ | | 95 | 21 | | 73 | 66 | 67 | 13 |
| Marker panel | | | 0.80 (0.76–0.83) | <10^-4^ | | 95 | 32 | | 80 | 69 | 71 | 20 |
| Marker Panel + PSA | | | 0.85 (0.81–0.88) | <10^-4^ | | 95 | 41 | | 84 | 72 | 74 | 26 |
| Combined^#^ | | | 0.87 (0.84–0.90) | <10^-4^ | 95 | | | 38 | 83 | 71 | 73 | 24 |

| **Validation cohort** | | |  | | |  | |  | |  | |  | |  | |  | |  |
| --- | --- | --- | --- | --- | --- | --- | --- | --- | --- | --- | --- | --- | --- | --- | --- | --- | --- | --- |
|  | | AUC (95% CI) | | | P | | Sen (%)* | | Spe (%) | | NPV (%) | | PPV (%) | | Accuracy (%) | | Bx avoided (%)^§^ | |
|  | **Model I: Benign vs Cancer** (Marker number in model=26) | | | | | | | | | | | | | | | | | |
| 5 clinical factors^†^ | | | | 0.75 (0.70–0.80) | | <10^-4^ | 95 | | 13 | | 72 | | 54 | | 55 | | 7 | |
| Marker panel | | | | 0.87 (0.83–0.91) | | <10^-4^ | 95 | | 34 | | 87 | | 60 | | 65 | | 18 | |
| Marker panel + PSA | | | | 0.88 (0.84–0.92) | | <10^-4^ | 95 | | 26 | | 84 | | 58 | | 62 | | 14 | |
| Combined^#^ | | | | 0.89 (0.86–0.93) | | <10^-4^ | 95 | | 47 | | 90 | | 66 | | 72 | | 24 | |
|  | **Model II: (Benign + VLR/LR) vs (FIR + UIR + HR/VHR + mPC)** (Marker number in model=24) | | | | | | | | | | | | | | | | | |
| 5 clinical factors^†^ | | | | 0.81 (0.76–0.86) | | <10^-4^ | 95 | | 16 | | 81 | | 16 | | 50 | | 10 | |
| Marker panel | | | | 0.93 (0.90–0.96) | | <10^-4^ | 95 | | 74 | | 95 | | 74 | | 83 | | 45 | |
| Marker Panel + PSA | | | | 0.94 (0.92–0.97) | | <10^-4^ | 95 | | 71 | | 95 | | 72 | | 82 | | 47 | |
| Combined^#^ | | | | 0.95 (0.93–0.97) | | <10^-4^ | 95 | | 75 | | 95 | | 75 | | 84 | | 47 | |
|  | **Model III: (Benign + VLR/LR + FIR) vs (UIR + HR/VHR + mPC)** (Marker number in model=26) | | | | | | | | | | | | | | | | | |
| 5 clinical factors^†^ | | | | 0.84 (0.80–0.89) | | <10^-4^ | 95 | | 33 | | 93 | | 44 | | 55 | | 23 | |
| Marker panel | | | | 0.88 (0.84–0.92) | | <10^-4^ | 95 | | 62 | | 96 | | 58 | | 74 | | 43 | |
| Marker Panel + PSA | | | | 0.92 (0.89–0.95) | | <10^-4^ | 95 | | 65 | | 96 | | 60 | | 76 | | 45 | |
| Combined^#^ | | | | 0.93 (0.90–0.96) | | <10^-4^ | 95 | | 67 | | 97 | | 62 | | 78 | | 47 | |
|  | **Model GS: (Benign + GS <7) vs (GS** ≥ **7)** (Marker number in model=22) | | | | | | | | | | | | | | | |  | |
| 5 clinical factors^†^ | | | | 0.78 (0.72–0.83) | | <10^-4^ | 95 | | 14 | | 85 | | 39 | | 44 | | 9 | |
| Marker panel | | | | 0.89 (0.86–0.93) | | <10^-4^ | 95 | | 68 | | 97 | | 64 | | 78 | | 43 | |
| Marker Panel + PSA | | | | 0.91 (0.88–0.94) | | <10^-4^ | 95 | | 69 | | 97 | | 65 | | 79 | | 44 | |
| Combined^#^ | | | | 0.91 (0.88–0.94) | | <10^-4^ | 95 | | 73 | | 97 | | 67 | | 81 | | 46 | |

P: *p* value for AUC (null hypothesis: AUC = 0.5); Sen: sensitivity; Spe: specificity; NPV: negative predictive value; PPV: positive predictive value; Bx: biopsy; CI: confidence interval; *sensitivity set at 95% for clinical relevance; ^§^The percent biopsy avoided was calculated after the cohort was normalized to the original risk group composition of the entire cohort enrolled during the study period; ^†^: 5 clinical risk factors; ^#^: metabolite marker panel plus 5 clinical risk factors. Tables 2 and 3 show the similar statistics at 90% sensitivity.

**Table S4**: Performance of the four predictive models in a subgroup with age more than or equal to 70 years (validation, 90% sensitivity)

|  | | AUC (95% CI) | P | Sen (%)* | Spe (%) | NPV (%) | PPV (%) | Accuracy (%) | Bx avoided (%)^§^ |
| --- | --- | --- | --- | --- | --- | --- | --- | --- | --- |
|  | **Model I: Benign vs Cancer** (Marker number in model=26) | | | | | | | | |
| 5 clinical factors^†^ | | 0.78 (0.71–0.86) | <10^-4^ | 90 | 28 | 70 | 61 | 63 | 15 |
| Marker panel | | 0.91 (0.86–0.95) | <10^-4^ | 90 | 65 | 85 | 76 | 79 | 34 |
| Marker panel + PSA | | 0.92 (0.88–0.96) | <10^-4^ | 90 | 78 | 87 | 84 | 85 | 41 |
| Combined^#^ | | 0.94 (0.90–0.97) | <10^-4^ | 90 | 84 | 88 | 87 | 88 | 44 |
|  | **Model II: (Benign + VLR/LR) vs (FIR + UIR + HR/VHR + mPC)** (Marker number in model=24) | | | | | | | |  |
| 5 clinical factors^†^ | | 0.84 (0.77–0.90) | <10^-4^ | 90 | 51 | 85 | 64 | 70 | 32 |
| Marker panel | | 0.94 (0.89–0.98) | <10^-4^ | 90 | 81 | 90 | 82 | 86 | 51 |
| Marker panel + PSA | | 0.96 (0.92–0.99) | <10^-4^ | 90 | 92 | 91 | 92 | 91 | 58 |
| Combined^#^ | | 0.96 (0.93–0.99) | <10^-4^ | 90 | 92 | 91 | 92 | 91 | 58 |
|  | **Model III: (Benign + VLR/LR + FIR) vs (UIR + HR/VHR + mPC)** (Marker number in model=26) | | | | | | | |  |
| 5 clinical factors^†^ | | 0.86 (0.80–0.92) | <10^-4^ | 90 | 56 | 89 | 59 | 70 | 39 |
| Marker panel | | 0.88 (0.82–0.93) | <10^-4^ | 90 | 72 | 91 | 70 | 80 | 50 |
| Marker panel + PSA | | 0.94 (0.91–0.98) | <10^-4^ | 90 | 85 | 93 | 81 | 88 | 59 |
| Combined^#^ | | 0.94 (0.91–0.97) | <10^-4^ | 90 | 84 | 93 | 80 | 87 | 59 |
|  | **Model GS: (Benign + GS <7) vs (GS** ≥ **7)** (Marker number in model=22) | | | | | | | |  |
| 5 clinical factors^†^ | | 0.82 (0.75–0.89) | <10^-4^ | 91 | 38 | 85 | 51 | 60 | 24 |
| Marker panel | | 0.93 (0.88–0.97) | <10^-4^ | 91 | 82 | 92 | 78 | 86 | 52 |
| Marker panel + PSA | | 0.95 (0.92–0.99) | <10^-4^ | 91 | 88 | 93 | 84 | 89 | 56 |
| Combined^#^ | | 0.94 (0.91–0.98) | <10^-4^ | 91 | 85 | 93 | 82 | 88 | 54 |

P: *p* value for AUC (null hypothesis: AUC = 0.5); Sen: sensitivity; Spe: specificity; NPV: negative predictive value; PPV: positive predictive value; Bx: biopsy; CI: confidence interval; GS: Gleason score; *sensitivity set at 90% for clinical relevance; ^§^The percent biopsy avoided was calculated after the cohort was normalized to the original risk group composition of the entire cohort enrolled during the study period; ^#^metabolite marker panel plus serum PSA. ^†^: 5 clinical risk factors; ^#^: metabolite marker panel plus 5 clinical risk factors.

**Table S5**: Performance of the four predictive models in a subgroup with PSA level less than 10 ng/ml (validation, 90% sensitivity)

|  | | AUC (95% CI) | P | Sen (%)* | Spe (%) | NPV (%) | PPV (%) | Accuracy (%) | Bx avoided (%)^§^ |
| --- | --- | --- | --- | --- | --- | --- | --- | --- | --- |
|  | **Model I: Benign vs Cancer** (Marker number in model=26) | | | | | | | | |
| 5 clinical factors^†^ | | 0.66 (0.58–0.74) | 10^-4^ | 90 | 18 | 74 | 43 | 48 | 9 |
| Marker panel | | 0.82 (0.76–088) | <10^-4^ | 90 | 36 | 85 | 50 | 59 | 19 |
| Marker panel + PSA | | 0.82 (0.75–0.88) | <10^-4^ | 90 | 25 | 81 | 46 | 52 | 13 |
| Combined^#^ | | 0.83 (0.77–0.89) | <10^-4^ | 90 | 45 | 88 | 53 | 64 | 23 |
|  | **Model II: (Benign + VLR/LR) vs (FIR + UIR + HR/VHR + mPC)** (Marker number in model=24) | | | | | | | |  |
| 5 clinical factors^†^ | | 0.70 (0.70–0.79) | <10^-4^ | 90 | 16 | 81 | 29 | 36 | 10 |
| Marker panel | | 0.91 (0.86–0.95) | <10^-4^ | 90 | 75 | 95 | 58 | 79 | 47 |
| Marker panel + PSA | | 0.90 (0.86–0.95) | <10^-4^ | 90 | 74 | 95 | 56 | 78 | 47 |
| Combined^#^ | | 0.91 (0.87–0.95) | <10^-4^ | 90 | 75 | 95 | 58 | 79 | 47 |
|  | **Model III: (Benign + VLR/LR + FIR) vs (UIR + HR/VHR + mPC)** (Marker number in model=26) | | | | | | | |  |
| 5 clinical factors^†^ | | 0.72 (0.61–0.83) | 10^-4^ | 90 | 13 | 87 | 19 | 27 | 9 |
| Marker panel | | 0.89 (0.84–0.93) | <10^-4^ | 90 | 80 | 98 | 50 | 82 | 56 |
| Marker panel + PSA | | 0.85 (0.77–0.92) | <10^-4^ | 90 | 63 | 97 | 36 | 68 | 44 |
| Combined^#^ | | 0.85 (0.78–0.92) | <10^-4^ | 90 | 63 | 97 | 36 | 68 | 44 |
|  | **Model GS: (Benign + GS <7) vs (GS** ≥ **7)** (Marker number in model=22) | | | | | | | |  |
| 5 clinical factors^†^ | | 0.67 (0.57–0.77) | 10^-3^ | 90 | 16 | 86 | 25 | 34 | 10 |
| Marker panel | | 0.89 (0.84–0.93) | <10^-4^ | 90 | 74 | 96 | 52 | 78 | 47 |
| Marker panel + PSA | | 0.88 (0.83–0.93) | <10^-4^ | 90 | 72 | 96 | 49 | 76 | 46 |
| Combined^#^ | | 0.88 (0.83–0.93) | <10^-4^ | 90 | 72 | 96 | 50 | 77 | 46 |

P: *p* value for AUC (null hypothesis: AUC = 0.5); Sen: sensitivity; Spe: specificity; NPV: negative predictive value; PPV: positive predictive value; Bx: biopsy; CI: confidence interval; GS: Gleason score; *sensitivity set at 90% for clinical relevance; ^§^The percent biopsy avoided was calculated after the cohort was normalized to the original risk group composition of the entire cohort enrolled during the study period; ^#^metabolite marker panel plus serum PSA. ^†^: 5 clinical risk factors; ^#^: metabolite marker panel plus 5 clinical risk factors.
